# Current Progress on Epidemiology, Diagnosis, and Treatment of Sporotrichosis and Their Future Trends

**DOI:** 10.3390/jof8080776

**Published:** 2022-07-26

**Authors:** Anderson Messias Rodrigues, Sarah Santos Gonçalves, Jamile Ambrósio de Carvalho, Luana P. Borba-Santos, Sonia Rozental, Zoilo Pires de Camargo

**Affiliations:** 1 Laboratory of Emerging Fungal Pathogens, Department of Microbiology, Immunology, and Parasitology, Discipline of Cellular Biology, Federal University of São Paulo (UNIFESP), Sao Paulo 04023062, Brazil; jamileambrosio@hotmail.com (J.A.d.C.); zpcamargo1@gmail.com (Z.P.d.C.); 2 Department of Medicine, Discipline of Infectious Diseases, Federal University of São Paulo (UNIFESP), Sao Paulo 04023062, Brazil; 3 Infectious Diseases Postgraduate Program, Center for Research in Medical Mycology, Federal University of Espírito Santo (UFES), Vitoria 29043900, Brazil; sarahunifesp@yahoo.com.br; 4 Cell Biology and Parasitology Program, Institute of Biophysics Carlos Chagas Filho, Federal University of Rio de Janeiro (UFRJ), Rio de Janeiro 21941902, Brazil; luana.p.borba@gmail.com (L.P.B.-S.); rozental@biof.ufrj.br (S.R.)

**Keywords:** *Sporothrix brasiliensis*, *Sporothrix schenckii*, *Sporothrix globosa*, sporotrichosis, implantation mycosis, subcutaneous mycosis, epidemiology, treatment, antifungal, diagnosis

## Abstract

Sporotrichosis, a human and animal disease caused by *Sporothrix* species, is the most important implantation mycosis worldwide. *Sporothrix* taxonomy has improved in recent years, allowing important advances in diagnosis, epidemiology, and treatment. Molecular epidemiology reveals that *S. brasiliensis* remains highly prevalent during the cat-transmitted sporotrichosis outbreaks in South America and that the spread of *S. brasiliensis* occurs through founder effects. *Sporothrix globosa* and *S. schenckii* are cosmopolitan on the move, causing major sapronoses in Asia and the Americas, respectively. In this emerging scenario, one-health approaches are required to develop a creative, effective, and sustainable response to tackle the spread of sporotrichosis. In the 21st century, it has become vital to speciate *Sporothrix*, and PCR is the main pillar of molecular diagnosis, aiming at the detection of the pathogen DNA from clinical samples through multiplex assays, whose sensitivity reaches remarkably three copies of the target. The treatment of sporotrichosis can be challenging, especially after the emergence of resistance to azoles and polyenes. Alternative drugs arising from discoveries or repositioning have entered the radar of basic research over the last decade and point to several molecules with antifungal potential, especially the hydrazone derivatives with great in vitro and in vivo activities. There are many promising developments for the near future, and in this review, we discuss how these trends can be applied to the *Sporothrix*-sporotrichosis system to mitigate the advance of an emerging and re-emerging disease.

## 1. A Brief Introduction to the System *Sporothrix*-Sporotrichosis

Sporotrichosis is a subcutaneous mycosis caused by the dimorphic fungus *Sporothrix schenckii* and related species, which are found worldwide in vegetation, decaying organic matter, *Sphagnum* moss, and soil [1]. Sporotrichosis is transmitted through traumatic inoculation of *Sporothrix* propagules into skin tissue [2]. The classical transmission route refers to sapronosis (i.e., environment to warm-blooded vertebrate host). Therefore, it is an occupational mycosis usually associated with trauma during outdoor work in gardeners, farmers, extractivist, and florists, among others. The alternative route of infection is related to horizontal animal transmission, mainly affecting domestic cats and armadillos [3,4]. In the cat-transmitted sporotrichosis, these animals spread the disease through scratches and bites or direct contact with their secretions to other cats, causing epizootics, or directly to humans (zoonosis) [5].

Most cases of human sporotrichosis manifest in the skin and subcutaneous tissues. The disease may vary according to the immune status of the infected host, with the lymphocutaneous form being the most common manifestation (~80% of cases) [6]. The fungus spread to bones and viscera is uncommon and occurs more frequently in immunosuppressed patients, especially in AIDS [7,8]. Pulmonary sporotrichosis, resulting from the inhalation of fungal propagules (conidia or yeasts), is uncommon [9].

Cats are the most susceptible hosts to contamination by *Sporothrix* and commonly develop the most severe forms of the disease, which can progress to death [10]. Multiple ulcerative lesions are usually observed in the cephalic region, mainly in the nose and paw region, due to feline behavior that involves scratching and biting during fights [10,11,12]. Sporotrichosis is higher among adult male cats, without owners, and those not neutered [10]. Different from what occurs in lesions in humans, a high number of yeasts can be observed in felines [13].

Spontaneous cure of human and animal sporotrichosis is rare, and treatment with antifungals is indispensable for most patients. Although localized sporotrichosis is readily treated, managing osteoarticular sporotrichosis, disseminated visceral forms, and feline sporotrichosis is laborious [2,14,15].

**Figure 1:**
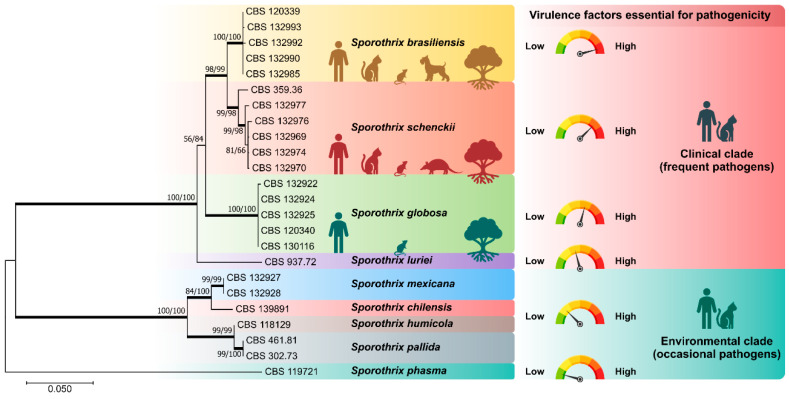
Phylogenetic analysis of the main members of medical relevance in the genus *Sporothrix* using sequences from the partial calmodulin-encoding gene (exons 3–5) and the ITS region (ITS1/2+5.8s). In the clinical clade, *S. brasiliensis* is highly virulent for the warm-blooded vertebrate host, followed by *S. schenckii*, *S. globosa*, and *S. luriei*. In the environmental clade, *S. chilensis*, *S. humicola*, *S. mexicana*, and *S. pallida* are occasional pathogens with mild-pathogenic potential to mammals. *Sporothrix phasma*, a species with no virulence to mammals, was used as an outgroup in the phylogenetic analysis. Numbers close to the branches represent bootstraps values (ML/NJ).

For over a century, *S. schenckii sensu lato* was described as the sole agent of human and animal sporotrichosis [16,17]. However, advances in molecular taxonomy revealed that it is not a monotypic taxon [18]. *Sporothrix* comprises approximately 53 species [15], including *S. brasiliensis*, *S. schenckii*, *S. globosa*, and *S. luriei*, forming a clade of clinical interest as they are frequently recovered from cases of sporotrichosis. The other *Sporothrix* species are embedded in the environmental clade and show little or no virulence to the warm-blooded vertebrate host [19]. Strictly environmental *Sporothrix* species are often associated with soil, insects, and plants. However, we highlight the members of the *S. pallida* complex (*S. chilensis*, *S. gemella*, *S. humicola*, *S. mexicana*, *S. pallida*, *S. palmiculminata*, *S. protea-sedis*, and *S. stylites*) which include soil-inhabitants fungi with mild-pathogenic potential for humans and animals (Figure 1) [20].

The differential pathogenicity in *Sporothrix* may be related to the efficiency in the temperature-induced morphological transition. Thermal dimorphism is an important morphological adaptation for infection, shared with other human pathogens, phylogenetically distant in the Onygenales and Eurotiales [21]. *Sporothrix* species nested in the clinical clade are ‘professional’ thermodimorphic fungi responding more efficiently to thermal stimuli. In addition, *S. brasiliensis* express important virulence attributes such as thermotolerance, adhesins, and melanin [22], being the most virulent species in murine models such as BALB/c [23,24], C57BL/6 [25], and OF-1 mice [26]. Such exacerbated virulence in animals is also observed in the human host, and *S. brasiliensis* is associated with atypical [27,28,29] and more severe forms of the disease, including disseminated skin infection in immunocompetent hosts and systemic disease [2,29,30,31].

## 2. Trends in the Epidemiology of *Sporothrix* Species

Sporotrichosis is a cosmopolitan mycosis whose etiological agents are constantly on the move. Species of clinical interest are not evenly distributed worldwide, and many are associated with different transmission routes [32]. No official statistics show the burden of human and animal sporotrichosis globally, but only case series denounce the problem. Therefore, it is a fact that sporotrichosis has classically been a mysterious disease from an epidemiological point of view.

Notoriously, the history of sporotrichosis shows repeated manifestations in the form of outbreaks and epidemics. The most famous epidemic occurred in the mid-1940s in South Africa, where more than 3000 native Bantu miners were infected with *Sporothrix* growing in the soil and the supporting timbers of the Witwatersrand gold mines [33,34,35]. Recently, to a lesser extent, a new case series was described in South Africa, showing that the fungus can persist in nature for decades. In South Africa, sapronotic transmission of the disease predominates, where *S. schenckii s. str.* is the main agent identified by molecular methods [36,37,38,39,40,41]. Animal sporotrichosis is rare in Africa [42], and environmental isolation shows the presence of members of the *S. pallida* complex [36]. Data on the occurrence of sporotrichosis on the African continent are scarce, being reported mainly in Madagascar [43], Zimbabwe [44], Nigeria [45], and Sudan [46]. In these areas, there is no correlation between the incidence of sporotrichosis and the HIV/AIDS epidemic. Despite a discreet series, the reports from the 1940s were fundamental for understanding the sapronotic route of the disease, clarifying, for the first time, the ecoepidemiological aspects of sporotrichosis [33,34,35].

On the Asian continent, epidemiological data arise mainly from Japan, China, India, and Malaysia, where there is a predominance of cases of human sporotrichosis due to *S. globosa*. Human sporotrichosis is endemic in India, occurring with high prevalence in the northern sub-Himalayan region, from Himachal Pradesh in the northwest to Assam and West Bengal in the east [47,48,49,50,51]. Historically, sporotrichosis was very common in Japan between the 1940s and 1980s, with significant remission since then [52,53,54,55]. In this country, molecular epidemiology was significantly influenced by RFLPs and PCR-RFLPs analysis of mtDNA, which revealed two main clades, groups A and B [54,55,56,57,58]. Currently, the reinterpretation of epidemiological data in light of taxonomic changes in *Sporothrix* confirms that *S. globosa* (group B) was the main agent of human sporotrichosis in Japan in the 1980s, followed by *S. schenckii s. str.* (group A) [59,60].

The incidence of human sporotrichosis in China is among the highest globally [61,62,63,64,65]. Cases are concentrated in the northeast region of China, in an area with a temperate continental monsoon climate, including Jilin, Liaoning, and Heilongjiang provinces [64,65,66,67]. Interestingly, there is a higher incidence of cases during the winter, possibly associated with contamination of the home environment with wood, twigs, and sticks used as an important energy matrix for cooking and heating [65,68]. Therefore, similar to epidemics in Africa, in Asia, sporotrichosis is a sapronosis whose main transmission route is the traumatic inoculation of plant material. However, unlike what happens in Africa, the etiological agent is *S. globosa* [3]. The exception to the rule is Malaysia, where *S. schenckii s. str.* can cause epizootics in domestic cats, increasing zoonotic transmission levels [69,70,71].

Human sporotrichosis was common on the European continent at the beginning of the last century. The cases emerged mainly in France and were richly reported in the literature [72,73,74]. However, the disease has decreased its incidence considerably since then, with rare case reports mainly from the United Kingdom, Spain, and Italy [18,75,76,77,78,79]. Notwithstanding, with the introduction of *S. brasiliensis* in England [80], our attention should be focused on the evolution of the number of cases in the coming years.

In Australia, sporadic reports of human sporotrichosis do not exceed a few hundred infections. Australian cases are generally associated with *S. schenckii s. str.* and *S. globosa* following a sapronotic route, mainly in Queensland, New South Wales, and Western Australia [81,82,83]. In these areas, outbreaks of sporotrichosis attributed to environmental sources such as hay are not uncommon. Feline sporotrichosis is rare in Australia, as described in the mid-1980s [84]. New cases have now been associated with *S. pallida* in cats, an even rarer association [85].

Sporotrichosis is relatively common in the Americas. In the USA, where the disease was first described in 1898 [16], *S. schenckii s. str.* causes illness in professionals linked to agricultural activities such as rose gardeners and farmers. The largest reported outbreak in the USA occurred in 1988 and affected 84 patients in 15 states exposed to the fungus in mosses of the genus *Sphagnum*, used in gardening procedures [86,87]. In the USA, between 2000 and 2013, 1471 hospitalizations were reported in patients with opportunistic conditions such as HIV/AIDS, immune-mediated inflammatory diseases, and chronic obstructive pulmonary disease [88].

In Latin America, sporotrichosis emerges as the most common implantation mycosis [89,90], with areas of high endemicity in Brazil [91], Colombia [92], Peru [93,94], and Venezuela [95]. However, we noticed significant differences that reflect on the species transmitted and on the route of transmission of the disease. For example, the sapronotic route of sporotrichosis is common throughout Latin America, where *S. schenckii s. str.* and *S. globosa* are spread through contact with fungal propagules present in the environment. On the other hand, the zoonotic route of sporotrichosis is more common in Brazilian territory, where cat-transmitted sporotrichosis is the main type of infection for humans, dogs, and other cats, and *S. brasiliensis* is the major agent in these cases [5].

**Figure 2:**
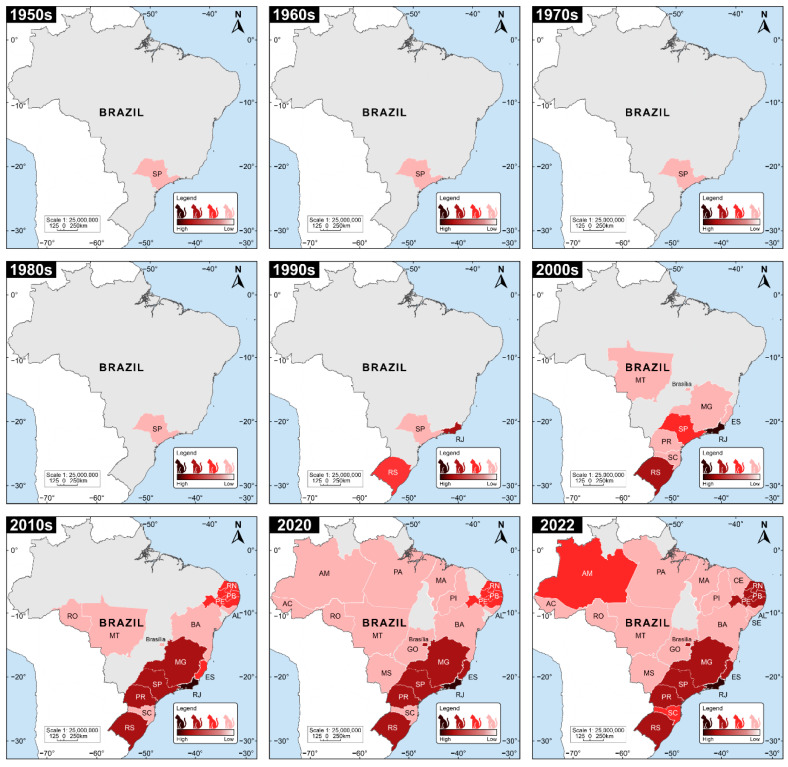
Temporal evolution of feline sporotrichosis cases in Brazil between 1950 and 2022. The current scenario of sporotrichosis shows signs of frank expansion. The map was drawn based on case reports available on the literature [5,10,12,15,31,32,68,91,98,99,100,101,105,108,114,115,116,117,118,119,120,121,122,123,124,125,126,127,128,129,130,131,132,133,134,135,136,137,138,139,140,141,142,143,144,145,146,147,148,149,150,151,152,153].

Case reports of human sporotrichosis occur in 25 of the 26 Brazilian states [3,91,96,97,98,99,100]. However, due to the emergence of sporotrichosis in cats, there is a marked temporal variation concerning the succession of species involved in transmissions (Figure 2). Before the 1990s, the classical sapronotic transmission of human sporotrichosis prevailed, similar in Latin American countries [91]. After the 1990s, with the entry of the domestic cat into the sporotrichosis transmission chain, it is possible to detect a considerable increase in epizootic manifestations in felines and zoonotic transmission to humans. This scenario has the metropolitan region of Rio de Janeiro as its epicenter, and between the 1990s and 2000s we observed a gradual spread of the epidemic to other states in the South and Southeast regions. Recently, in full expansion, we described the emergence of *S. brasiliensis* in the northeast region of the country [91,101], mainly in the states of Pernambuco [102], Paraíba [103], and the Rio Grande do Norte [31,104,105]. Interestingly, in these areas of feline sporotrichosis, *S. schenckii s. str.* is no longer the main transmitted species, and *S. brasiliensis* becomes the main agent during the cat-transmitted sporotrichosis events (Figure 2).

Judging from a public health point of view, the major drawback in the above scenario is the absence of a national notification system to report disease cases. Since 2011, the notification of sporotrichosis has been mandatory in the State of Rio de Janeiro, but not in other Brazilian states, with rare specific exceptions at regional and municipal levels [13,100,106,107,108].

In general terms, the geographic fluctuation of sporotrichosis agents is fascinating. *Sporothrix globosa* is the predominant molecular type in Asia. *S. schenckii s. str.* is highly prevalent in Australia, South Africa, western South and Central America, and North America. *Sporothrix brasiliensis* is highly prevalent in Brazil. Among all medically relevant *Sporothrix* species, *S. brasiliensis* has the greatest potential for geographic dispersal. In areas where it occurs, *S. brasiliensis* easily outperforms other clinically relevant species due to feline transmission [101]. To date, Argentina and Paraguay have reported the occurrence of *S. brasiliensis* in humans and cats outside Brazil [109,110,111], and there are suspected cases in Bolivia, Colombia, and Panama [112,113]. Recently, a zoonotic case was reported in the UK after a veterinarian treated a cat with sporotrichosis imported from Brazil [80]. In the absence of official epidemiological data, case reports show the importance of the disease, its local and regional escalation, and the urgent need to establish sanitary barriers to mitigate the advance of *S. brasiliensis* and the cat-transmitted sporotrichosis.

**Figure 3:**
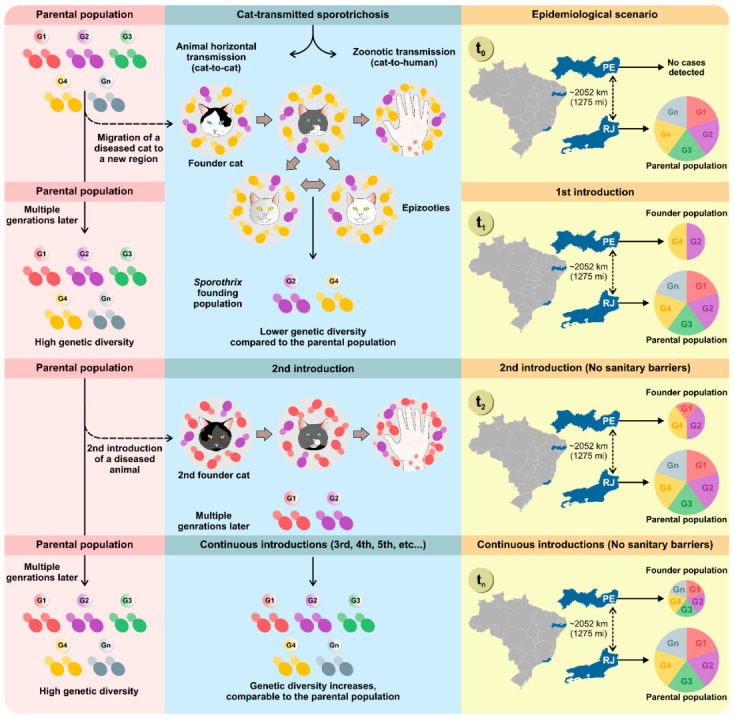
Founder effect events explain the expansion dynamics of cat-transmitted sporotrichosis. The molecular studies developed by de Carvalho et al. [101] offer new bases for proposing public policies to mitigate sporotrichosis. The *S. brasiliensis* genotypes (e.g., G1, G2, G3, G4, G5, Gn) infect cats living in the metropolitan region of Rio de Janeiro and are considered the parental population. Eventually, a sick cat infected with a single or a group of genotypes (e.g., G2 and G4) is taken to a new area (e.g., Pernambuco), where it will establish a founder population, transmitting *S. brasiliensis* to other cats (epizootics) or humans (zoonoses). A study of genetic diversity at time one (t1) will reveal that the founder population has less genetic diversity when compared to the parental population. However, the absence of sanitary barriers and the continuous exchange of diseased animals taken by their tutors from the parental-to-founder population will gradually (t2, t3, t4, tn) reconstitute the genetic diversity in the founding population and accelerate the pace of diversification.

In feline sporotrichosis, it is generally accepted that a single diseased cat introduced to a new location can trigger an outbreak that will quickly evolve into an epidemic. A new dissemination area may include locations as close as a neighborhood, a new city, or even more distant areas such as other states. Introducing diseased animals to new areas has occurred repeatedly within the natural history of cat-transmitted sporotrichosis. The metropolitan region of Rio de Janeiro is described as the possible center of origin, from where the disease initially spread to other border states in the southeastern region (e.g., São Paulo, Espírito Santo, and Minas Gerais) and later to the southern region. (e.g., Paraná). The most recent migration event occurred towards the Brazilian northeast in mid-2015 (Figure 3).

De Carvalho and colleagues reported that cat-transmitted sporotrichosis progresses through founder effects (Figure 3) [101]. In population genetics, a founder effect refers to the reduction in genetic variability that occurs when a small group of individuals not genetically representative of the parental population migrates to a new area and establishes a new population. Over time, the resulting new subpopulation will have genotypes and phenotypic characteristics similar to the founding individual, which may differ greatly from the parent population. Therefore, a founder effect may explain, for example, the low genetic diversity found during the initial outbreaks of cat-transmitted sporotrichosis. However, the absence of sanitary barriers and the constant introduction of sick animals (parental population → founder population) to new areas can reconstitute genetic diversity in the founder population, leading to comparable genetic diversity.

Cats adapt to a wide range of environments, and in general, the roaming area of domestic cats (0.02–10 ha) overlaps with the human residential area, where they can more easily secure food [154,155,156]. Therefore, the only viable hypothesis to justify the detection of a genotype from the parental population of Rio de Janeiro in areas as remote as the state of Pernambuco in the northeast region (>2000 km) is the introduction of sick cats via humans who migrate with their pets, since sporotrichosis is not a disease of direct person-to-person transmission or even a zooanthroponosis. This hypothesis may also explain the introduction of sick cats in other South American countries, such as Argentina and Paraguay [109,110,111], or even the European continent, as recently reported in England [80]. Establishing sanitary barriers to contain the migration of sick felines is a fundamental measure for controlling the expansion of *S. brasiliensis* (Figure 3).

The recent outbreaks in Brazil due to cat-transmitted sporotrichosis and the widespread expansion in South America are important reminders of how human and non-human health are essentially connected. Animals are the source of 70% of emerging and re-emerging infectious disease threats to human health and more than half of all recognized human pathogens [157,158,159,160]. We observed that the recent entry of the domestic cat into the transmission chain of sporotrichosis associated with the emergence of *S. brasiliensis*, a more virulent species adapted to animal transmission, produced a significant revolution in the classical epidemiological pattern [101], confirming that such threats are dynamic [161].

Although the absence of official and reliable data makes it difficult to measure the problem, cat-transmitted sporotrichosis is responsible for a significant burden of sporotrichosis in Brazil [15]. Geoepidemiological analyses of zoonotic sporotrichosis cases in Rio de Janeiro, Brazil, reveal that the social determinants of the disease are linked to social vulnerability. The disease mainly affects women (25 to 59 years old), especially in socioeconomically disadvantaged neighborhoods of Rio de Janeiro, expressed by low per capita income and deficient supply of treated water to households [162]. This shows that sporotrichosis can be aggravated in scenarios of greater social vulnerability [160,163], a regretful development that tends to be repeated in other areas of the country [133,164].

**Figure 4:**
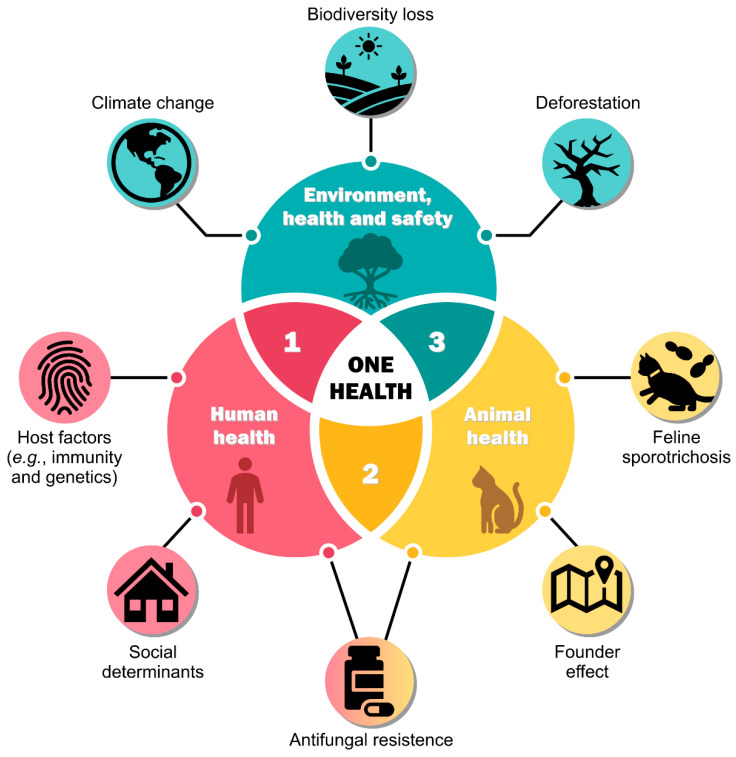
A one-health approach to mitigating the spread of cat-transmitted sporotrichosis considers human health (1), animal health (2), and environmental health and safety (3).

Therefore, a public health problem that encompasses human, animal, and environmental health issues requires solutions based on one-health approaches (Figure 4). One-health approaches consider the interactions among different spheres of global health to develop a creative, effective, and sustainable response. Therefore, interdisciplinary research is mandatory, as is interventionist practice at local, national, and international levels, involving public managers, physicians, veterinarians, biologists, public and animal health authorities, environmental health agents, and microbiologists, among other allies.

The current Brazilian environmental scenario results from climate change, intense deforestation, and biodiversity loss [165]. The Intergovernmental Panel on Climate Change’s sixth assessment report reveals that a 1.5 °C rise in global temperature would result in a 100–200% increase in the population affected by floods in Colombia, Brazil, and Argentina, 300% in Ecuador, and 400% in Peru (medium confidence) [166]. Higher temperatures, heavy rainfall, and flooding are associated with an increase in emerging zoonotic diseases [166,167]. Medically relevant *Sporothrix* can be detected in the soil of endemic areas where it remains for years [168,169], and it is interesting to hypothesize that water from floods and inundations, a phenomenon increasingly common in Brazil, may also promote the diffusion of *Sporothrix* propagules in the soil.

In this chaotic scenario, we incorporate the fact that in endemic areas, cats with sporotrichosis are often buried directly in the soil, producing latent foci of the pathogen. Soil functions as the main reservoir of fungal propagules and certainly does not act as a passive reservoir. The advance of deforestation in the Amazon, Cerrado, and Atlantic Forest is worrying, leading to biodiversity loss in these biomes. It is known that soil microbial composition can be altered due to deforestation, reflecting co-occurrence patterns among microorganism taxa, leading to ecological imbalance [170,171]. For example, soil amoebas (e.g., *Acanthamoeba castellanii*) rapidly change the composition of the bacterial community in the soil [172], and it is well known that many of these protozoa interact with *Sporothrix*, predating the microorganism in the soil [173,174]. Therefore, it is expected that environmental stresses (e.g., higher temperatures, humidity, pH, etc.) that affect *A. castellanii* in the soil [175], leading to alterations in biodiversity, may reflect population imbalances in the fluctuation of *Sporothrix* species (Figure 4).

These scenarios demonstrate that one-health solutions are complex to implement in their totality, yet they are crucial to combat the spread of emerging *Sporothrix* species (Figure 4) [176].

## 3. Trends in the Diagnosis of Sporotrichosis

Sporotrichosis can be diagnosed through a correlation of clinical, epidemiological, and laboratory data [32]. The early and accurate laboratory diagnosis of sporotrichosis is of substantial importance since the clinical aspect of cutaneous lesions can mimic other dermatologic manifestations, such as mycobacteriosis, actinomycosis, American tegumentary leishmaniasis, blastomycosis, cryptococcosis, paracoccidioidomycosis, among others [2]. In addition, ulcerative lesions can mimic pyoderma gangrenosum [177]. Clinical suspicion and the patient’s epidemiological context are key to assembling this puzzle and thus promptly establishing the diagnosis.

The diagnosis of sporotrichosis is based on the isolation and identification of *Sporothrix* in culture, cytopathology, histopathology, sporotrichosis skin test, serology, immunohistochemistry, and molecular techniques [10,15,78,116,178]. Moreover, complementary laboratory tests such as blood count and biochemical profile should be requested in systemic forms. Anemia, neutrophilic leukocytosis, gammopathies, and hypoalbuminemia are commonly observed [2,7].

### 3.1. Mycological Test

Currently, the reference method for sporotrichosis diagnosis remains the isolation and identification of microorganisms in culture media and characterization of the agent by morphological parameters [179,180,181,182]. Although it has been widely used in clinical routine, the method sensitivity is not 100%, which can generate false-negative results due to contamination of the samples with bacteria and anemophilous fungi and inadequate transport of the material [181,182,183]. While considered a low-cost diagnosis, it is noteworthy that it is not suitable for diagnosing atypical and extracutaneous forms of the disease [184].

Depending on the clinical form and laboratory approaches, several biological samples can be investigated. The most frequent specimens are tissue fragments, serosanguineous exudates, purulent secretion, scraping of hyperkeratotic crusts, aspirate of lymph nodes, and organ fragments obtained during necropsy [5,185]. It must be emphasized that some care must be observed not to attenuate the technique sensitivity, such as material transport temperature, time storage, and appropriate clinical sample processing [186].

To ensure the fungus viability, some criteria should be pursued, including (i) seed the material as soon as possible in culture media; (ii) transport swabs on Stuart media; (iii) preserve tissue fragment biopsies in sterile saline solution (mycological tests) or formalin solution (histological examinations). Furthermore, the sooner the biological material is seeded in culture media, the greater the chances of recovery of the microorganism. Otherwise, amid eventualities, the clinical sample should be kept at 4 °C for a time less than 8–10 h [130].

The average growth time of *Sporothrix* spp. in mycelial form (25 °C) is 3–5 days to two weeks [187]. The isolates obtained can be accurately identified from positive cultures, and antifungal susceptibility testing in vitro and other assays can be performed [2]. Macroscopically, in media such as malt extract agar (MEA) or potato dextrose agar (PDA), the colonies start to grow as hyaline filamentous fungi and then turn brown to black after a few days, mainly in the colony’s center [118,188,189]. Sometimes the mycelium can grow entirely white, designated as an albino [118,188,189]. The *Sporothrix* colonies’ diameter is smaller, ranging from 19 to 41 mm, compared to other filamentous fungi, such as *Aspergillus fumigatus* (65–70 mm) [118,190]. Microscopically, the mycelial form is observed as septate, thin, branched, and hyaline hyphae, with conidiogenous cells arising from undifferentiated hyphae, forming conidia thick-walled (hyaline or brown), small (2–3 × 3–6µm), with a different arrangement, such as sympodial form, appearing in small groups of denticles in a slight apical dilation of the conidiophore or as sessile [118,188,189].

*Sporothrix* is a thermodimorphic fungus. To ensure its identification, it is recommended to stimulate the morphological transition. The fungus should be seeded in enriched media such as blood glucose-cysteine agar or Brain Heart Infusion agar (BHI) and subsequent incubation at 35–37 °C to obtain the yeast form [177,187]. The growth time is the same as the mycelial form. In some cases, the isolates grow slowly, so the fungi should be incubated for up to 30 days for the outcome [181,191,192]. In yeast form, the colonies are tan or cream-colored and smooth. Micromorphologically, it is possible to observe spindle-shaped and oval cells measuring 2.5–5 μm in diameter, like cigar-shaped buds on a narrow base [15,118,181,192].

Although the phenotypic characterization is not distinctly effective to speciate *Sporothrix*, it allows the presumptive identification of some species belonging to the clinical clade. Some characteristics, such as the color and shape of conidia, suggest some clinical species, such as *S. brasiliensis* and *S. schenckii s. str.* [78,193]. The latter predominates more elongated conidia, and some isolates showed triangular pigmented conidia, thereof being characteristic of the species. Concerning *S. brasiliensis*, the conidia are mostly more globose dematiaceous conidia, yet they may have the presence or absence of melanin [2,78,118]. Micromorphological characteristics may also hint at identifying *S. chilensis*, *S. mexicana*, and *S. pallida*, commonly environmental species, with mid-pathogenic potential for humans [20,78]. All these aspects are subtle, and their variations can lead to errors in identifying the species [75,98,118,194].

Marimon et al. [78] and Rudramurthy et al. [51] demonstrated that physiological characteristics such as growth rate, thermotolerance, and sugar assimilation might be helpful in the differentiation of morphologically similar species embedded in the clinical clade. Besides, some studies imply that medically relevant *Sporothrix* can be distinguished from environmental, according to the analysis of growth rates and thermotolerance [195]. Although not entirely elucidated, clinical strains probably have specific characteristics acquired during their evolution that undoubtedly contributed to their pathogenicity [195].

Toward accurately speciating *Sporothrix*, the polyphasic approach is most advisable for assembled morphological, physiological, molecular, and ecological characteristics [19,98]. Whereas culture remains a reference in the sporotrichosis diagnosis, the delayed results may impact the severity and compromise the disease treatment, decreasing the probability of cure in cats and humans and improving the transmission risk.

### 3.2. Direct Microscopic, Cytopathological, and Histopathological Examinations

Biological material obtained from human skin lesions and tissue fragments has a low fungal burden, and the yeast size (2–6 μm) hinders their visualization in the direct microscopic examination (DME) of fresh material. Thus, DME of samples treated with potassium hydroxide solution (KOH, 10–30%) or with fast staining techniques should not be recommended [185,196,197]. However, the DME shows better positivity in immunosuppressed patients [2,198]. The DME specificity and sensitivity of the tissue samples are still unknown, as most investigators consider this tool inefficacious [177].

According to Orofino-Costa et al. [2], purulent secretion imprints or biopsies stained with Giemsa increase the test’s sensitivity in humans. In extracutaneous forms, the DME sensitivity is even lower; occasionally, it is possible to observe the fungal structures in cigar or shuttles forms [199].

Cytopathological examination stained with periodic acid-Schiff (PAS) or Gomori-methenamine silver (GMS), the aspiration puncture of the lesions, especially in the extracutaneous and disseminated forms, eventually allows the observation of granuloma of epithelioid cells, asteroid bodies, and yeast cells [177]. In 20% of cases, asteroid bodies may be observed in the center of the granuloma [196,200,201]. Asteroid bodies are globular or oval yeast cells surrounded by radiated eosinophilic material (Splendore-Hoeppli reaction), including antibodies (IgM and IgG), with the role of defense against phagocytes [32,202].

Findings of asteroid bodies in the stained cytopathological examination are very unpredictable since it depends on the staining method and displays minor reproducibility. It is not a sporotrichosis pathognomonic structure, as it may be observed in other granulomatous and infectious diseases [177,185,196,203]. Gram, Giemsa, PAS, and GMS may be successfully used in disseminated manifestations [200,201].

**Figure 5:**
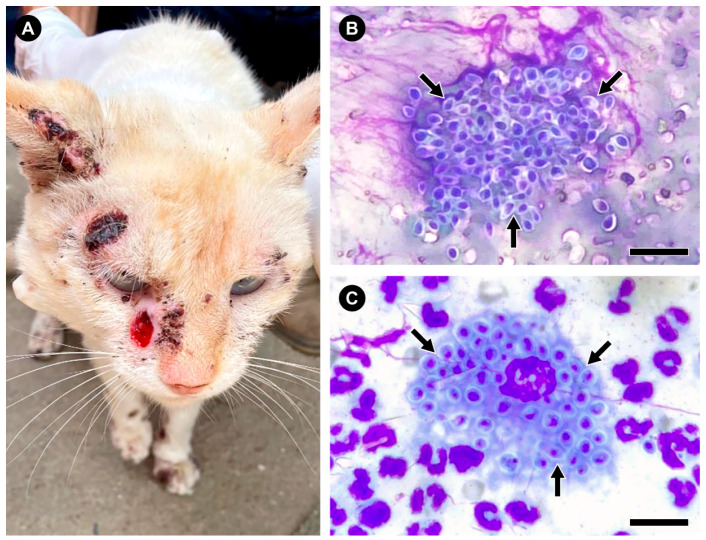
The diagnosis of feline sporotrichosis can employ simple, fast, and inexpensive methods such as the quick Panoptic method. (**A**) Clinical aspect of feline sporotrichosis with ulcerated lesions in the cephalic region of a cat from the state of Espírito Santo, Brazil. (**B**,**C**) Feline macrophages infected with numerous *S. brasiliensis* yeasts cells (arrows), stained using the quick Panoptic method. Bar = 15 µm.

Conversely, Gram, quick Panoptic, Wright, Giemsa, or Rosenfeld cytopathological staining techniques are more sensitive in animals, particularly felines (Figure 5A) [116]. The cytopathological examination from exudates and skin lesions shows a high fungal burden, making it possible to observe *Sporothrix* yeast cells that range from being rounded, oval, or cigar-shaped, surrounded by a transparent, capsule-like halo, as seen in *Cryptococcus* spp. and *Histoplasma* spp. [10,32,179]. These structures may be disposed of inwardly in macrophages, neutrophils, and multinucleated or free giant cells [130,131]. Concerning feline sporotrichosis, asteroid bodies are uncommon [204].

The quick Panoptic method, a Romanowsky-type staining technique like Diff-Quik, has become relatively common in veterinary clinics due to its practicality, low cost, and great return (Figure 5B,C). A staining kit profits around 1000 slides [116,205]. This diagnosis has a sensitivity of 52.6% to 95% in cats compared to culture, the reference method [116,120,206]. However, for non-ulcerated or low exudative lesions, treatment with antifungals in high doses seems to interfere negatively with this method’s sensitivity [116,120]. In the last years, the feline sporotrichosis laboratory diagnosis starts with cytology by imprinting the lesions in glass slides and isolating the fungus in culture [207]. In this scenario, an old-fashioned method such as cell block cytology achieves an impressive 97.5% sensitivity when diagnosing feline sporotrichosis during outbreaks and epidemics [208].

As in cytopathology, the histopathological findings in human samples may be nonspecific, only signaling the human sporotrichosis diagnosis. The paucity of fungal structures is also similar [183,204,209]. The histopathological pattern is associated with granulomatous and pyogenic reactions, which may lodge with epidermal hyperplasia (with or without ulceration), papillomatous acanthosis, hyperkeratosis, intraepidermal microabscess, and fungal elements such as yeast cells and asteroid bodies [196,209]. In approximately 50% of cases, the yeasts may be visible in tissue smears stained with PAS and GMS [196]. Briefly, the granuloma caused by *Sporothrix* may show three distinct zones: (i) center with abscesses or necrosis (central zone); (ii) area with granulomatous inflammation constituted by giant cells (tuberculoid zone); and (iii) lymphocytes and plasm cells, with granulation tissue and fibrosis (syphiloid zone) [209]. The inflammatory infiltrates are best observed by hematoxylin-eosin (HE) staining [177,196]. In patients with AIDS, the histopathological findings are uncommon; the inflammatory response is lower, and there is no presence of asteroid bodies. However, the amount of yeast is profuse [210].

### 3.3. Serology

The first serological tests for sporotrichosis diagnosis were performed in 1910 using tube agglutination (TA) and complement fixation (FC) tests [211]. The tests using precipitins were described in 1947, employing a polysaccharide antigen [212]. In 1973, Blumer et al. [213], besides testing these methods, also evaluated immunodiffusion (ID), slide agglutination test (SLA), and indirect fluorescent antibody (IFA). At that time, the SLA and ID methods were the most specific. SLA had the highest sensitivity (94%), proving to be an easy-to-perform technique that generates quick results and is highly recommended for clinical routine.

Sporotrichin, an antigenic complex consisting of a peptide-rhamnomannan, is obtained from a crude extract of the mycelial phase of *S. schenckii sensu lato*. This antigenic fraction was developed for intradermal skin reactions to measure the degree of immunity or receptivity of the individual with suspected disease, determining first contact with the fungus without developing the disease [212,214]. Sporotrichin has a long history in surveillance studies depicting areas of high endemicity [6,215].

The absence of a standardized or commercial antigen impacts the serological diagnosis of sporotrichosis in Europe and the USA [6,216]. Nevertheless, sporotrichin effectiveness in highly endemic regions ranges from 89 to 96%, making it an interesting additional test [216,217,218,219,220]. Bonifaz et al. [221] reported that false-positive results might be attributed to patients in constant contact with the agent, such as those who live in endemic areas or cases in which the patient preserves immunological memory. However, false-negative points correspond to patients with different immunosuppression degrees.

Many attempts have been made to adopt serological tests such as sporotrichosis diagnostic methods, given that they are fast, highly accurate, and not invasive [222]. The fungal cell wall antigens and anti-cell wall antibodies became the main target of studies searching to develop serological tools that are more sensitive and specific [222,223,224].

It is well known that *S. schenckii* displays a mixture antigen complex, including peptide-rhamnomannan, a cell wall glycoconjugate (CWPR) of the yeast phase of the fungus. This structure can be fractionated by affinity chromatography on Sepharose 4B with concanavalin A (Con A), generating two fractions: one binding to Con A (SsCBF) and the other non-binding to Con A (SsNBF). The fraction bound to Con A is relevant for serological diagnosis [225,226]. Besides, techniques such as immunoblot, fluorescent antibodies, counterimmunoelectrophoresis (CIE), double immunodiffusion (DID), and enzyme-linked immunosorbent assays (ELISA) have been quoted as auspicious [227,228].

A study by Penha and Lopes-Bezerra [213], using the ELISA method, evaluated 35 patients with the sporotrichosis cutaneous form, and SsCBF showed 100% specificity with the investigated sera. Subsequent studies have also demonstrated high levels of sensitivity and specificity for the various clinical presentations of human sporotrichosis, including extracutaneous, lymphocutaneous, fixed, and disseminated forms [222,229].

Bernardes-Engemann et al. [184] validated the ELISA test applying purified antigenic fraction SsCBF for the human sporotrichosis diagnosis by detecting the anti-SsCBF immunoglobulin G (IgG) antibody fraction. Thus, 177 serum samples from different clinical forms were evaluated. The investigators observed high specificity (82%) and sensitivity (89%) with a reproducibility of 98%, including for emerging species such as *S. brasiliensis*, and may be made disposable for routines of the health services.

One year after that, Alvarado et al. [227] evaluated the potential of the crude antigen obtained from the mycelial form of *S. schenckii s. str.* in serum from sporotrichosis patients by applying different methods, including DID, CIE, and ELISA. The assays were validated using serum from disease patients such as paracoccidioidomycosis, histoplasmosis, leishmaniasis, tuberculosis, lupus, and serum from healthy patients. Investigators achieved 100% sensitivity and >98% specificity for all tests.

Fernandes et al. [225] evaluated ELISA’s test using SsCBF and an exoantigen for the domestic cats’ serological assay with suspected sporotrichosis. The authors reported 90% sensitivity and 96% specificity for SsCBF. Excellent results were also reached by Rodrigues et al. [122], testing different antigen types obtained from crude extracts of *S. schenckii s. str.* and *S. brasiliensis* yeast cells. The results suggest that the ELISA technique with distinct antigens may be applied in diagnosing feline sporotrichosis. Furthermore, all the antigens studied reacted similarly, with no significant difference in titer. Therefore, the results generated by antigens of the different etiological agents should probably not interfere, likely because antigenic epitopes are shared and well conserved between *S. schenckii* and *S. brasiliensis* [24,230].

Baptista et al. [217] recently validated felines serology; however, it has been available only in private clinics and laboratories. The authors modified the SsCBF-ELISA test for human serological diagnosis and the quantification of IgG antibodies for all clinical forms of feline sporotrichosis.

Recombinant *Sporothrix* antigens have also been studied as diagnostic markers [224], and in 2019, Martinez-Alvarez and colleagues [231] evaluated an ELISA test for the human sporotrichosis detection from a recombinant glycoprotein obtained from the cell wall of *S. schenckii*, Gp70. Nonetheless, the findings were not as promising for the diagnosis compared to the SsCBF antigen [231].

The hyperepidemic worsening of feline and human sporotrichosis has reached proportions in different regions and countries. Public policies should be established to contain the disease, and new diagnostic methods should be developed to prevent the spread of sporotrichosis. Therefore, serological methods can qualitatively and quantitatively evaluate the condition, generating fast results with high levels of specificity and sensitivity. It would already be a big step, as it would directly impact the diagnosis of the disease and the diagnostic screening and therapeutic monitoring of human and animal sporotrichosis, including those who develop atypical and severe forms [232].

### 3.4. Molecular Diagnosis

For the last decades, the classical identification of the species belonging to the *Sporothrix* genus was based only on phenotypic methods [22,87,233,234,235]. Although these methods supply reduced cost, they are laborious, time-consuming between collection and final diagnosis, and do not allow to speciate *Sporothrix* since they have overlapping morphological and physiological features [15,95,98,181,192,194]. It is essential to highlight that the phenotypic plasticity in *Sporothrix* may exist even intraspecifically [20,78,193].

With the advance and improvement of molecular tools, it is possible to identify, reclassify, and recognize new species of fungi, allowing a greater understanding of the biology of these microorganisms [236,237]. Regarding the molecular identification of *Sporothrix*, some critical points should be considered; among them, we highlight the type of sample (e.g., soil, biopsy, isolated culture, exudate, vegetable), the cost of technique, the time that the process is executed, sensitivity/specificity, and DNA quality [238,239]. The advances in molecular diagnosis of sporotrichosis were recently reviewed by de Carvalho et al. [240].

The polymerase chain reaction (PCR) and its variants are widely used in human and feline sporotrichosis diagnosis [241,242]. It is possible to carry out DNA amplification using several molecular markers and different methods depending on the proposed objective from clinical specimens and fungi obtained from growth in culture. Kano et al. [242] pioneered the non-culture-dependent PCR technique. From human tissue, they identified *S. schenckii sensu lato* using chitin synthase I (*CHS1*) as a target gene to amplify DNA. In 2003, Kano and collaborators [243] applied this same technique in biopsy samples obtained from six patients with human sporotrichosis, confirmed by histopathology and mycological findings. The generated DNA fragments showed 99% similarity with *S. schenckii* reference strain.

**Figure 6:**
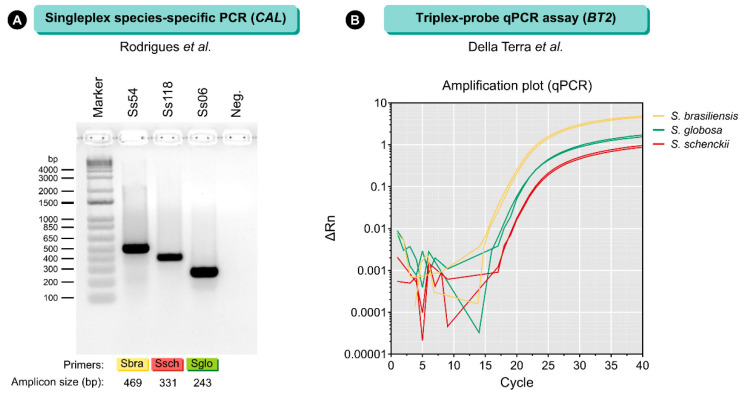
The molecular diagnosis of sporotrichosis relies on PCR. (**A**) Species-specific PCR is a fast and inexpensive method that combines conventional PCR and agarose gel electrophoresis to detect *Sporothrix* DNA, mainly in vitro culture samples [96]. Eventually, species-specific PCR is used for detection from clinical samples [249]. Although it has great specificity, the method has low sensitivity (up to 10–100 fg of DNA). (**B**) For the rapid and accurate diagnosis of human and feline sporotrichosis, a multiplex qPCR assay was developed for the simultaneous detection and speciation of *S. brasiliensis*, *S. schenckii*, and *S. globosa* from cultured DNA and clinical samples (up to 3 copies of the target) [238]. Ss54 = *S. brasiliensis*; Ss118 = *S. schenckii*; Ss06 = *S. globosa*.

After that, other molecular tools with the potential to detect the *Sporothrix* DNA directly from environmental samples (soils, plants, decaying wood, tree bark) and clinical specimens obtained from humans and animals (biopsy, skin lesion, aspirate of abscess, exudate swabs, and pus) were proposed. The most frequently used techniques have been species-specific PCR (Figure 6A), nested PCR, restriction fragment length polymorphism (RFLP), and qPCR (quantitative real-time PCR). Among the different targets, the most common are 18S rRNA [241,244], Large Subunit (LSU), internal transcribed spacer (ITS) [245], β-tubulin (*BT2*) [238], calmodulin (*CAL*) [96,246,247,248], and mitochondrial DNA genes [59,60]. Nevertheless, it is worth mentioning that few techniques allow for detecting *Sporothrix* DNA from clinical samples and identifying it down to a species level.

Della-Terra et al. [238] developed and evaluated a multiplex qPCR assay for sporotrichosis diagnosis exploiting polymorphisms found in the β-tubulin gene (Figure 6B). Samples of cat lesions and environmental samples spiked with *S. brasiliensis*, *S. schenckii*, and *S. globosa* propagules were used to reveal the feasibility of the method to detect *Sporothrix* DNA. High specificity (100%) and sensitivity (98.6%) were reported [238]. Moreover, the technique did not show cross-reaction with other pathogenic fungi, human, feline, or murine DNA, allowing the identification of all major *Sporothrix* species in a single tube. The key advantages of the multiplex qPCR assay include decreasing amount of reagents, reduction of the process steps, greater sensitivity when compared to culture [114], species-specific PCR [96,249], rolling circle amplification (RCA) [247], and other qPCR assays [245,250].

Regarding identifying and recognizing cryptic species belonging to *Sporothrix*, the reference method is DNA sequencing (Sanger) followed by phylogenetic analysis. In addition to amplification and partial sequencing of the ITS region (ITS1/2+5.8s), protein-coding loci such as *BT2* [18], translation elongation factor (EF-1α) [20], and *CAL* [18,78,193] should be included [189]. Nowadays, the region between exons 3 and 5 of the *CAL* gene is the primary marker for identifying clinical species for this genus [19,20,62,79,91,95,98,99,109,114,251,252].

Techniques like *CAL*-RFLP [246], RCA [247], and species-specific PCR [96] can also identify *Sporothrix* down to species level. The major difference among methods is that RCA and species-specific PCR can detect *Sporothrix* DNA, whereas *CAL*-RFLP was designed for strains isolated in culture.

DNA fingerprint methods such as amplified fragment length polymorphisms (AFLPs) have demonstrated broad applicability in speciating pathogenic fungi and describing genetic diversity. AFLP is widely used in evolutionary, population, epidemiological, and conservation studies of different taxa [253]. AFLP’s main advantage is the simultaneous assessment of several loci, randomly distributed throughout the genome, without prior knowledge of the DNA sequence. This makes the AFLP singularly helpful for species with no genomic knowledge, and a powerful tool to be used to explore *Sporothrix* genetic diversity, answer questions related to the structure of the population, transmission routes, intra and interspecific variability, as well as modes of recombination and reproduction, among many other biological issues [254,255,256].

In the last decades, mass profiles of ribosomal proteins generated by Matrix-Assisted Laser Desorption/Ionization Time-of-Flight Mass Spectrometry (MALDI-ToF MS) have been a powerful tool in yeast identification in routine clinical laboratories [257,258]. In recent years, MALDI-ToF MS databases have been built and expanded to identify filamentous fungi; for the technique to be reliable, rapid, and economical, databases must be accurate [259,260]. The method was standardized to speciate *Sporothrix* isolates growing in vitro during the yeast phase, which allows the recognition of *S. brasiliensis*, *S. schenckii*, *S. globosa*, *S. luriei*, and members of the *S. pallida* complex. Furthermore, speciation by MALDI-ToF or DNA sequencing methods are in full agreement [259]. However, the greatest limitation of MALDI-TOF relies on the need for prior culture. Only a few reports in the literature use this methodology to speciate *Sporothrix*. Matos et al. [261] constructed an in-house database enriched with spectra generated from reference *Sporothrix* strains, enabling the identification of an isolate from *S. brasiliensis* obtained from a patient with subconjunctival infiltrative injury in a right eye.

Despite the relevance of molecular diagnostic tools, unfortunately, these techniques are not widely available in health services, being restricted to research laboratories and reference centers, thus negatively impacting the diagnosis of the disease.

## 4. Trends in the Treatment of Sporotrichosis

The treatment choice for sporotrichosis depends on the disease’s clinical form, the host’s immunological status, and the species *Sporothrix* involved. The most virulent species (*S. brasiliensis*, *S schenckii*, and *S. globosa*) exhibit different susceptibility profiles to antifungals; thus, the response to therapy can be variable [262].

Sporotrichosis was first described in 1898 when specific antifungal drugs were unavailable, and potassium iodide (KI) was used for several infectious and non-infectious diseases. KI saturated solution has been used for treating sporotrichosis since 1903 [2]. KI has immunomodulatory activity; it can suppress the production of toxic intermediates from oxygen by the polymorphonuclear leukocytes and, therefore, exert its anti-inflammatory effect. KI’s ability to directly destroy microorganisms is still a matter of speculation. Notwithstanding, reports in the literature suggest that when *Sporothrix* yeasts are exposed to increasing drug concentrations, cell lysis occurs through the release of lysosomal enzymes [263]. Recent studies show that KI inhibits biofilm development in *Sporothrix* [264]. The main adverse events of KI are metallic taste and nausea, followed by an acneiform eruption. Nowadays, its use in humans has been replaced by itraconazole, but due to its low cost, it is still used to treat cutaneous human forms and felines, in association or not with itraconazole [14,265,266].

**Figure 7:**
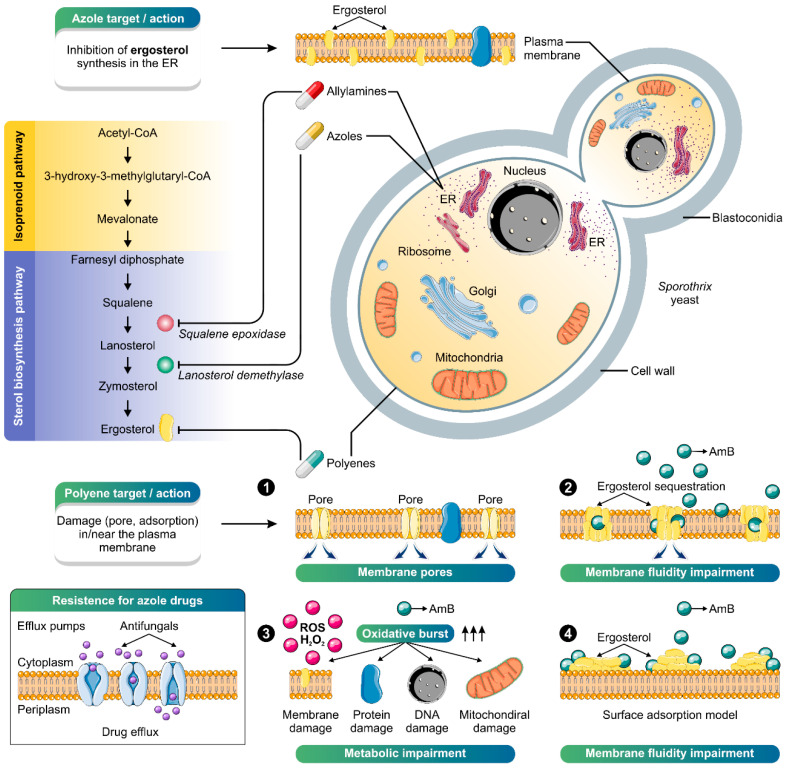
The main antifungal agents used in treating sporotrichosis and their cellular targets are depicted. Azoles (e.g., itraconazole) and allylamines (e.g., terbinafine) are fungistatic drugs that slow fungal growth; the azoles by inhibiting cytochrome P-450-dependent synthesis of ergosterol (purple chart: sterol biosynthesis pathway) and the allylamines by competitive inhibition of squalene epoxidase, blocking the conversion of squalene to lanosterol. Amphotericin B is a fungicidal drug whose main target is the ergosterol molecule, producing pores in the plasma membrane (1), leading to the leakage of cytoplasmic material, sequestering (2), absorbing or extracting ergosterol from the membrane (4). Oxidative damage is reported as an alternative mode of action of AmB (3). The main adaptation that can lead to resistance in *Sporothrix* species is the increased expression of efflux pumps, especially to azoles, although other mechanisms may be involved. The illustration was partially based on Servier Medical Art elements and licensed under a Creative Commons Attribution 3.0 Unported License. ER: endoplasmic reticulum; ROS: reactive oxygen species.

Itraconazole started to be used in sporotrichosis treatment in the late 1980s, during the advent of the triazoles generation. Itraconazole is the drug of choice due to its effectiveness, safety, and posologic convenience for lymphocutaneous and cutaneous sporotrichosis. In Brazil, it is also used in animal treatment [15,189,266]. Itraconazole is a fungistatic drug that inhibits the synthesis of ergosterol, the main sterol from the fungus cell membrane (Figure 7) [267]. Depending on the disease severity and the host’s immunological status, the therapeutic dose may range from 100 to 400 mg/day [188]. Although it has good efficacy, treatment with itraconazole can cause several side effects and interact with more than 200 other drugs, inducing adverse events or lacking effectiveness [268]. The main adverse effects reported are headaches and gastrointestinal disorders. It is also hepatotoxic, teratogenic, and embryotoxic, and may not be used in patients with liver diseases or pregnant women.

Another inhibitor of ergosterol synthesis used for sporotrichosis treatment is terbinafine (Figure 7). This allylamine treats cutaneous form in humans when itraconazole or KI is not tolerated or cannot be used [269]. It is available in 125 and 250 mg tablets, facilitating pediatric administration. The recommended dose is 250 mg/day, but it may increase to 500 mg/day for adults [2].

In severe life-threatening cases, amphotericin B (deoxycholate or, preferably, liposomal, because such formulation has fewer adverse effects) is recommended until clinical improvement has been achieved, when it should be replaced with itraconazole. Amphotericin B is a polyene antifungal drug developed in the 1950s. There are four models proposed for the polyene mode of antifungal action: (i) the pore-forming model, (ii) the surface adsorption model, (iii) the sterol sponge model, and (iv) the oxidative damage model. In every suggested model, the binding of the polyene with ergosterol is essential to its antifungal effect [270] (Figure 7). The total cumulative dose recommended for amphotericin B ranges from 1 to 3 g. Although effective and acts with fungicidal properties, treatment with amphotericin B is not recommended for cutaneous and lymphocutaneous sporotrichosis because of its high toxicity and the inconvenience of intravenous administration [7,8].

Although the sporotrichosis treatment is mainly based on the prescription of the antifungals described above, other commercial drugs can also inhibit the *Sporothrix* growth in vitro. Table 1 shows the activities of different commercial antifungals according to the minimum inhibitory concentration (MIC) values obtained in vitro by the broth microdilution methods described by the Clinical and Laboratory Standards Institute [271,272]. Most of these antifungals are not used in treating sporotrichosis and are only used in the topical treatment of other fungal infections. Amphotericin B, itraconazole, and terbinafine exhibit higher in vitro activity against *Sporothrix* cells, with MIC described in the literature as lower than 1 µg/mL (Table 1).

Little is known about in vivo activity of other azoles for sporotrichosis treatment (Table 1), except for voriconazole and posaconazole. Voriconazole exhibits modest efficacy, while posaconazole is effective against disseminated sporotrichosis murine models [273,274]. Although they have been used for other fungal infections, they are not currently used for sporotrichosis treatment.

Non-pharmacological measures are also used in treating sporotrichosis with good results, such as cryosurgery and thermotherapy. Cryosurgery is indicated when a slow itraconazole response is observed for the resolution of chronic lesions and when adverse effects lead to interruption of the antifungals [275]. Local hyperthermia and cryosurgery are safe and efficacy options for treating pregnant women with cutaneous sporotrichosis [275,276]. Photodynamic therapy could also be applied as a non-pharmacological treatment for sporotrichosis, considering the promising in vitro and in vivo studies; however, further clinical observation is still necessary [277].

In recent years, there has been an increase in therapeutic failures in treating sporotrichosis and reports of isolates with low sensitivity to itraconazole [97,267,278,279,280,281,282,283,284]. The decrease of itraconazole effectiveness against *S. brasiliensis* observed in clinical isolates from Brazil over the last years could be related to resistance mechanisms developed by this species, such as overexpression of efflux pumps (Figure 4). The increased expression of efflux pumps in the fungal membrane reduces the antifungal activity due to the extrusion of the drug, decreasing its effect. The overexpression of efflux pumps corresponds to the main mechanism of acquired resistance to azoles in fungi of medical relevance [285]. However, other resistance mechanisms could be related to *S. brasiliensis* decreasing susceptibility to itraconazole, such as melanin production and overexpression or mutation of the target enzyme [281]. On the other hand, studies investigating new molecules with anti-*Sporothrix* activity have increased.

We performed a retroactive literature search for studies published between 2012 and July 2022 using the databases Cortellis Drug Discovery Intelligence (https://www.cortellis.com/drugdiscovery/ (accessed on 14 July 2022) [268] and PubMed (https://pubmed.ncbi.nlm.nih.gov/ (accessed on 14 July 2022), with “*Sporothrix*” as the Keywords. We considered promising compounds with MIC against *Sporothrix* species less or equal to 4 µg/mL or 1 µM, as determined by Clinical and Laboratory Standards Institute protocols [271,272]. Based on these criteria, we found seventeen new molecules or repositionable drugs over the last ten years (Table 2). Most studies listed in Table 2 are Brazilian research papers highlighting the importance of this pathogen in Brazil. Only two of the seventeen papers are not from Brazilian groups.

Furthermore, twelve of these works were published in the last five years, reflecting the increase in studies using *Sporothrix* as a model due to its importance as a human pathogen. Besides, most compounds induce disruption of the cell membrane in *Sporothrix* cells. The MIC values show that farnesol and buparvaquone are the most promising compounds (Table 2). However, considering in vitro and in vivo approaches, the hydrazone derivative 4-bromo-N′-(3,5-dibromo-2-hydroxybenzylidene)-benzohydrazide (reported as D13 in the original study) exhibited the most interesting results, with good in vitro and in vivo activities [290].

## 5. Conclusions

The current scenario shows the emergence and re-emergence of sporotrichosis as a cosmopolitan mycosis whose etiological agents are in constant movement and associated with different transmission routes. Therefore, public policies should vary according to the epidemiological scenario, preferably using one-health strategies. Policies aimed at the human–environment interface are mandatory for areas where the sapronotic route prevails, driven by *S. globosa* and *S. schenckii s. str*. For areas where cat-transmitted sporotrichosis is emerging, policies aimed at the human–animal–environment interface are necessary (e.g., responsible animal ownership; limiting the number of cats per house; neutering campaigns; limiting cat access to the streets; cremation of dead cats). The invasive capacity of *S. brasiliensis* is impressive, becoming the predominant species shortly after its introduction into a new area. Such dynamics of the expansion of cat-transmitted sporotrichosis associated with *S. brasiliensis* occur through successive founder effect events. Therefore, the imposition of sanitary barriers preventing the free movement of sick animals is vital to tackle the geographic expansion of *S. brasiliensis* beyond the borders of Brazil.

In a rapidly expanding epidemic scenario, diagnostic tools need to keep up with the pace of the problem. Therefore, fast and accurate methods are important tools for laboratory diagnosis. In feline sporotrichosis, the quick Panoptic method is an efficient alternative for diagnosis given the high fungal load in the lesions, in addition to the robustness and low price. However, speciation will only be possible through the application of molecular tools. The pillar of the molecular diagnosis of sporotrichosis relies on PCR, with conventional assays that combine in vitro amplification and agarose gel electrophoresis (e.g., species-specific PCR) to multiplex reactions capable of real-time detecting mixed infections in a single tube (e.g., qPCR). Rapid diagnosis allows specific treatment, which positively impacts patients’ clinical outcomes and reduces transmission rates.

The choice of treatment for sporotrichosis depends on the triad, (i) the clinical form of the disease, (ii) the immune status of the host, and (iii) the *Sporothrix* species involved. The main challenge for the coming years is the sudden emergence of isolates resistant to itraconazole, the first choice to treat sporotrichosis. The good news is that a search in the Cortellis Drug Discovery Intelligence database revealed 17 new molecules or repositionable drugs in the last ten years, highlighting the hydrazone derivatives as a promising alternative based on great in vitro and in vivo activities.

All the scenarios contemplated above are interdependent and must be considered to mitigate the advance of the major mycosis of implantation worldwide.

## Figures and Tables

**Table 1:** In vitro antifungal activity against pathogenic *Sporothrix* species.

In Vitro Antifungal Activity ^a^				
	High (MIC ≤ 1 µg/mL)	Moderate (1 < MIC ≤ 4 µg/mL)	Low (MIC > 4 µg/mL)	Reference
**Polyenes**				
Amphotericin B				[262]
**Azoles**				
Albaconazole				[286]
Clotrimazole				[287]
Eberconazole				[286]
Fluconazole				[286]
Itraconazole				[262]
Isavuconazole				[288]
Ketoconazole				[262]
Miconazole				[286]
Posaconazole				[262]
Ravuconazole				[286]
Voriconazole				[262]
**Allylamines**				
Terbinafine				[262]
Naftifine				[289]
**Echinocandins**				
Anidulafungin				[286]
Caspofungin				[286]
Micafungin				[286]
**Pirimidine**				
Flucytosine				[286]

^a^ In vitro antifungal activity is determined according to MIC values obtained by the microdilution technique [271,272].

**Table 2:** New compounds, natural products, and repositionable drugs exhibited promising activity against *Sporothrix* spp. in the last ten years.

Group	Compound	Minimum Inhibitory Concentration ^a^	Antifungal Effect or Mechanism of Action	Reference
Hydrazone derivatives	22-hydrazone-imidazolin-2-yl-chol-5-ene-3β-ol	0.01–0.5 μg/mL	Inhibition of ergosterol biosynthesis	[291]
	4-bromo-N′-(3,5-dibromo-2-hydroxybenzylidene)-benzohydrazide	0.12–1 μg/mL	Inhibition of vesicular transport and cell cycle progression	[290]
Oxadiazole	N-[5-(4-chlorophenyl)-1,3,4-oxadiazol-2-yl]-3-(trifluoromethyl)benzamide	0.25–0.5 μM	Cell membrane disruption and neutral lipid accumulation	[292]
Alkylphospholipid analogc	TCAN26	0.25–2 μg/mL	Cell membrane disruption	[293]
Pentathiepin	23	0.5–1 μg/mL	Unknown	[294]
Benzisothiazolone	1.9	0.5 μg/mL	Apoptosis induction	[295]
Metal complex	Zn(itraconazole)2Cl_2_	0.08 μM	Unknown	[296]
	Zn(ketoconazole)2(Ac)2⋅H_2_O	0.125 μM	Unknown	[297]
	[Cu(PPh3)2(ketoconazole)2]NO_3_	0.006 μM	Unknown	[298]
Naphthoquinone derivative	2,5-dichloro-3,6-bis(4-methylpiperazin-1-yl)cyclohexa-2,5-diene-1,4-dione	1.56 µg/mL	Unknown	[299]

Natural products	farnesol	0.003–0.222 μg/mL	Unknown	[300]

Repositionable drugs	Miltefosine	1–2 µg/mL	Cell membrane disruption	[278]
		0.25–2 μg/mL		[301]
	Iodoquinol	0.5–1 μM	Cell membrane disruption	[302]
	Buparvaquone ^b^	0.005–0.16 μg/mL	Mitochondrial dysfunction	[303]
	Ibuprofen	0.03–0.5 μg/mL ^c^	Cell membrane disruption and ROS accumulation	[292]
	Pentamidine	0.06–0.25 μg/mL	DNA intercalation	[304]

^a^ MIC range or MIC mean [271,272].; ^b^ veterinary use; ^c^ combined with itraconazole, amphotericin B, or terbinafine.

## Data Availability

The data presented in this study are available within the article.

## References

[1] Ramírez-Soto M., Aguilar-Ancori E., Tirado-Sánchez A., Bonifaz A. (2018). Ecological determinants of sporotrichosis etiological agents. J. Fungi.

[2] Orofino-Costa R.C., Macedo P.M., Rodrigues A.M., Bernardes-Engemann A.R. (2017). Sporotrichosis: An update on epidemiology, etiopathogenesis, laboratory and clinical therapeutics. An. Bras. De Dermatol..

[3] Rodrigues A.M., de Hoog G.S., de Camargo Z.P. (2016). *Sporothrix* species causing outbreaks in animals and humans driven by animal-animal transmission. PLoS Pathog..

[4] Rodrigues A.M., Bagagli E., de Camargo Z.P., Bosco S.M.G. (2014). *Sporothrix schenckii sensu stricto* isolated from soil in an armadillo’s burrow. Mycopathologia.

[5] Gremião I.D., Miranda L.H., Reis E.G., Rodrigues A.M., Pereira S.A. (2017). Zoonotic epidemic of sporotrichosis: Cat to human transmission. PLoS Pathog..

[6] Bonifaz A., Vázquez-González D. (2013). Diagnosis and treatment of lymphocutaneous sporotrichosis: What are the options?. Curr. Fungal Infect. Rep..

[7] Kauffman C.A., Bustamante B., Chapman S.W., Pappas P.G. (2007). Clinical practice guidelines for the management of sporotrichosis: 2007 update by the Infectious Diseases Society of America. Clin. Infect. Dis..

[8] Kauffman C.A., Hajjeh R., Chapman S.W., Group M.S. (2000). Practice guidelines for the management of patients with sporotrichosis. Clin. Infect. Dis..

[9] Aung A.K., Teh B.M., McGrath C., Thompson P.J. (2013). Pulmonary sporotrichosis: Case series and systematic analysis of literature on clinico-radiological patterns and management outcomes. Med. Mycol..

[10] Gremião I.D., Menezes R.C., Schubach T.M., Figueiredo A.B., Cavalcanti M.C., Pereira S.A. (2015). Feline sporotrichosis: Epidemiological and clinical aspects. Med. Mycol..

[11] Pereira S.A., Gremião I.D.F., Menezes R.C., Zeppone Carlos I. (2015). Sporotrichosis in Animals: Zoonotic Transmission. Sporotrichosis: New Developments and Future Prospects.

[12] Pereira S.A., Gremião I.D., Kitada A.A., Boechat J.S., Viana P.G., Schubach T.M. (2014). The epidemiological scenario of feline sporotrichosis in Rio de Janeiro, State of Rio de Janeiro, Brazil. Rev. Da Soc. Bras. De Med. Trop..

[13] Schubach A., Schubach T.M., Barros M.B., Wanke B. (2005). Cat-transmitted sporotrichosis, Rio de Janeiro, Brazil. Emerg. Infect. Dis..

[14] Gremião I.D.F., Martins da Silva da Rocha E., Montenegro H., Carneiro A.J.B., Xavier M.O., de Farias M.R., Monti F., Mansho W., de Macedo Assunção Pereira R.H., Pereira S.A. (2021). Guideline for the management of feline sporotrichosis caused by *Sporothrix brasiliensis* and literature revision. Braz. J. Microbiol. Publ. Braz. Soc. Microbiol..

[15] Rodrigues A.M., Della Terra P.P., Gremiao I.D., Pereira S.A., Orofino-Costa R., de Camargo Z.P. (2020). The threat of emerging and re-emerging pathogenic *Sporothrix* species. Mycopathologia.

[16] Schenck B.R. (1898). On refractory subcutaneous abscesses caused by a fungus possibly related to the *Sporotricha*. Bull. Johns Hopkins Hosp..

[17] Hektoen L., Perkins C.F. (1900). Refractory subcutaneous abscesses caused by *Sporothrix schenckii*: A new pathogenic fungus. J. Exp. Med..

[18] Marimon R., Gené J., Cano J., Trilles L., Dos Santos Lazéra M., Guarro J. (2006). Molecular phylogeny of *Sporothrix schenckii*. J. Clin. Microbiol..

[19] de Beer Z.W., Duong T.A., Wingfield M.J. (2016). The divorce of *Sporothrix* and *Ophiostoma*: Solution to a problematic relationship. Stud. Mycol..

[20] Rodrigues A.M., Cruz Choappa R., Fernandes G.F., De Hoog G.S., Camargo Z.P. (2016). *Sporothrix chilensis* sp. nov. (Ascomycota: Ophiostomatales), a soil-borne agent of human sporotrichosis with mild-pathogenic potential to mammals. Fungal Biol..

[21] Sil A., Andrianopoulos A. (2014). Thermally dimorphic human fungal pathogens—Polyphyletic pathogens with a convergent pathogenicity trait. Cold Spring Harb. Perspect. Med..

[22] Fernandes G.F., dos Santos P.O., Amaral C.C., Sasaki A.A., Godoy-Martinez P., Camargo Z.P.d. (2009). Characteristics of 151 Brazilian *Sporothrix schenckii* isolates from 5 different geographic regions of Brazil: A forgotten and re-emergent pathogen. Open Mycol. J..

[23] Fernandes G.F., dos Santos P.O., Rodrigues A.M., Sasaki A.A., Burger E., de Camargo Z.P. (2013). Characterization of virulence profile, protein secretion and immunogenicity of different *Sporothrix schenckii sensu stricto* isolates compared with *S. globosa* and *S. brasiliensis* species. Virulence.

[24] Della Terra P.P., Rodrigues A.M., Fernandes G.F., Nishikaku A.S., Burger E., de Camargo Z.P. (2017). Exploring virulence and immunogenicity in the emerging pathogen *Sporothrix brasiliensis*. PLoS Negl. Trop. Dis..

[25] Almeida-Paes R., de Oliveira L.C., Oliveira M.M.E., Gutierrez-Galhardo M.C., Nosanchuk J.D., Zancopé Oliveira R.M. (2015). Phenotypic characteristics associated with virulence of clinical isolates from the *Sporothrix* complex. BioMed Res. Int..

[26] Arrillaga-Moncrieff I., Capilla J., Mayayo E., Marimon R., Mariné M., Gené J., Cano J., Guarro J. (2009). Different virulence levels of the species of *Sporothrix* in a murine model. Clin. Microbiol. Infect..

[27] de Macedo P.M., Sztajnbok D.C., Camargo Z.P., Rodrigues A.M., Lopes-Bezerra L.M., Bernardes-Engemann A.R., Orofino-Costa R. (2015). Dacryocystitis due to *Sporothrix brasiliensis*: A case report of a successful clinical and serological outcome with low-dose potassium iodide treatment and oculoplastic surgery. Br. J. Dermatol..

[28] Silva-Vergara M.L., de Camargo Z.P., Silva P.F., Abdalla M.R., Sgarbieri R.N., Rodrigues A.M., dos Santos K.C., Barata C.H., Ferreira-Paim K. (2012). Disseminated *Sporothrix brasiliensis* infection with endocardial and ocular involvement in an HIV-infected patient. Am. J. Trop. Med. Hyg..

[29] Almeida-Paes R., de Oliveira M.M., Freitas D.F., do Valle A.C., Zancope-Oliveira R.M., Gutierrez-Galhardo M.C. (2014). Sporotrichosis in Rio de Janeiro, Brazil: *Sporothrix brasiliensis* is associated with atypical clinical presentations. PLoS Negl. Trop. Dis..

[30] Nepomuceno Araujo M., Nihei C.H., Rodrigues A.M., Higashino H., Ponzio V., Campos Pignatari A.C., Barcellos M.A., Braga O., Duayer I.F. (2021). Case report: Invasive sinusitis due to *Sporothrix brasiliensis* in a renal transplant recipient. Am. J. Trop. Med. Hyg..

[31] do Monte Alves M., Pipolo Milan E., da Silva-Rocha W.P., Soares de Sena da Costa A., Araujo Maciel B., Cavalcante Vale P.H., de Albuquerque P.R., Lopes Lima S., Salles de Azevedo Melo A., Messias Rodrigues A. (2020). Fatal pulmonary sporotrichosis caused by *Sporothrix brasiliensis* in Northeast Brazil. PLoS Negl. Trop. Dis..

[32] Barros M.B., de Almeida Paes R., Schubach A.O. (2011). *Sporothrix schenckii* and sporotrichosis. Clin. Microbiol. Rev..

[33] Dangerfield L.F., Gear J. (1941). Sporotrichosis among miners on the Witwatersrand gold mines. S.A. Med. J..

[34] Pijper A., Pullinger B.D. (1927). An outbreak of sporotrichosis among South African native miners. Lancet.

[35] Brown R., Bowen J.W., Weintroub D., Cluver E.H., Buchanan G., Simson F.W., Lurie H.I., Du Toit C.J., Golberg L., Goedvolk C.J. Sporotrichosis infection on mines of the Witwatersrand. Proceedings of the Transvaal Mine Medical Officer’s Association.

[36] Govender N.P., Maphanga T.G., Zulu T.G., Patel J., Walaza S., Jacobs C., Ebonwu J.I., Ntuli S., Naicker S.D., Thomas J. (2015). An outbreak of lymphocutaneous sporotrichosis among mine-workers in South Africa. PLoS Negl. Trop. Dis..

[37] Vismer H.F., Hull P.R. (1997). Prevalence, epidemiology and geographical distribution of *Sporothrix schenckii* infections in Gauteng, South Africa. Mycopathologia.

[38] Nenoff P., Reinel D., Kruger C., Grob H., Mugisha P., Suss A., Mayser P. (2015). Tropical and travel-related dermatomycoses: Part 2: Cutaneous infections due to yeasts, moulds, and dimorphic fungi. Der Hautarzt Z. Fur. Dermatol. Venerol. Und Verwandte Geb..

[39] Callens S.F., Kitetele F., Lukun P., Lelo P., Van Rie A., Behets F., Colebunders R. (2006). Pulmonary Sporothrix schenckii infection in a HIV positive child. J. Trop. Pediatrics.

[40] Ponnighaus M., Grosser S., Baum H.P., Mischke D., Kowalzick L. (2003). Sporotrichosis as the cause of a leg ulcer. Der Hautarzt; Z. Fur Dermatol. Venerol. Und Verwandte Geb..

[41] Findlay G. (1985). Sporotrichosis research in the Transvaal-how it began 60 years ago. S. Afr. Med. J. Suid-Afrik. Tydskr. Vir Geneeskd..

[42] Dalis J.S., Kazeem H.M., Kwaga J.K., Kwanashie C.N. (2014). Severe generalized skin lesions due to mixed infection with *Sporothrix schenkii* and *Dermatophilus congolensis* in a bull from Jos, Nigeria. Vet. Microbiol..

[43] Rasamoelina T., Maubon D., Raharolahy O., Razanakoto H., Rakotozandrindrainya N., Rakotomalala F.A., Bailly S., Sendrasoa F., Ranaivo I., Andrianarison M. (2019). Sporotrichosis in the Highlands of Madagascar, 2013–2017. Emerg. Infect. Dis..

[44] Ross M.D., Gelfand M. (1978). Deep fungal infections in Rhodesia—A 10-year survey of histological material. Part I Cent. Afr. J. Med..

[45] Jacyk W.K., Lawande R.V., Tulpule S.S. (1981). Deep mycoses in West Africa: A report of 13 cases and review of the Nigerian literature. J. Natl. Med. Assoc..

[46] Gumaa S.A. (1978). Sporotrichosis in Sudan. Trans. R. Soc. Trop. Med. Hyg..

[47] Chakrabarti A., Bonifaz A., Gutierrez-Galhardo M.C., Mochizuki T., Li S. (2015). Global epidemiology of sporotrichosis. Med. Mycol..

[48] Sharma N.L., Mehta K.I., Mahajan V.K., Kanga A.K., Sharma V.C., Tegta G.R. (2007). Cutaneous sporotrichosis of face: Polymorphism and reactivation after intralesional triamcinolone. Indian J. Dermatol. Venereol. Leprol..

[49] Mehta K.I.S., Sharma N.L., Kanga A.K., Mahajan V.K., Ranjan N. (2007). Isolation of *Sporothrix schenckii* from the environmental sources of cutaneous sporotrichosis patients in Himachal Pradesh, India: Results of a pilot study. Mycoses.

[50] Verma S., Verma G.K., Singh G., Kanga A., Shanker V., Singh D., Gupta P., Mokta K., Sharma V. (2012). Sporotrichosis in Sub-Himalayan India. PLoS Negl. Trop. Dis..

[51] Rudramurthy S.M., Shankarnarayan S.A., Hemashetter B.M., Verma S., Chauhan S., Nath R., Savio J., Capoor M., Kaur H., Ghosh A.K. (2021). Phenotypic and molecular characterisation of *Sporothrix globosa* of diverse origin from India. Braz. J. Microbiol. Publ. Braz. Soc. Microbiol..

[52] Fukushiro R. (1984). Epidemiology and ecology of sporotrichosis in Japan. Zent. Bakteriol. Mikrobiol. Hyg. A.

[53] Ishizaki H., Kawasaki M. (2000). Molecular epidemiology of *Sporothrix schenckii*. Nihon Ishinkin Gakkai Zasshi.

[54] Takeda Y., Kawasaki M., Ishizaki H. (1991). Phylogeny and molecular epidemiology of *Sporothrix schenckii* in Japan. Mycopathologia.

[55] Suzuki K., Kawasaki M., Ishizaki H. (1988). Analysis of restriction profiles of mitochondrial DNA from Sporothrix schenckii and related fungi. Mycopathologia.

[56] Ishizaki H., Kawasaki M., Aoki M., Wu S., Lin J., Kim J.A., Won Y.H., Calvo C.R. (2004). Mitochondrial DNA analysis of *Sporothrix schenckii* from China, Korea and Spain. Nihon Ishinkin Gakkai Zasshi.

[57] Ishizaki H., Kawasaki M., Aoki M., Vismer H., Muir D. (2000). Mitochondrial DNA analysis of *Sporothrix schenckii* in South Africa and Australia. Med. Mycol..

[58] Ishizaki H., Kawasaki M., Aoki M., Matsumoto T., Padhye A.A., Mendoza M., Negroni R. (1998). Mitochondrial DNA analysis of Sporothrix schenckii in North and South America. Mycopathologia.

[59] Mochizuki H., Anzawa K., Mochizuki T. (2022). Genotyping of intraspecies polymorphisms of *Sporothrix globosa* using partial sequence of mitochondrial DNA. J. Derm..

[60] Kawasaki M., Anzawa K., Mochizuki T., Ishizaki H. (2012). New strain typing method with *Sporothrix schenckii* using mitochondrial DNA and polymerase chain reaction restriction fragment length polymorphism (PCR–RFLP) technique. J. Derm..

[61] Moussa T.A., Kadasa N.M., Al Zahrani H.S., Ahmed S.A., Feng P., Gerrits van den Ende A.H., Zhang Y., Kano R., Li F., Li S. (2017). Origin and distribution of *Sporothrix globosa* causing sapronoses in Asia. J. Med. Microbiol..

[62] Liu T.-T., Zhang K., Zhou X. (2014). Molecular identification of *Sporothrix* clinical isolates in China. J. Zhejiang Univ. Sci. B.

[63] Yu X., Wan Z., Zhang Z., Li F., Li R., Liu X. (2013). Phenotypic and molecular identification of *Sporothrix* isolates of clinical origin in Northeast China. Mycopathologia.

[64] Lv S., Hu X., Liu Z., Lin Y., Wu H., Li F. (2022). Clinical epidemiology of sporotrichosis in Jilin province, China (1990-2019): A series of 4969 cases. Infect. Drug Resist..

[65] Song Y., Li S.S., Zhong S.X., Liu Y.Y., Yao L., Huo S.S. (2013). Report of 457 sporotrichosis cases from Jilin province, northeast China, a serious endemic region. J. Eur. Acad. Derm. Venereol..

[66] Yao L., Song Y., Cui Y., Zhou J.F., Zhong S.X., Zhao D.Y., Li S.S. (2019). Pediatric sporotrichosis in Jilin province of China (2010-2016): A retrospective study of 704 cases. J. Pediatric Infect. Dis. Soc..

[67] Yao L., Song Y., Zhou J.F., Cui Y., Li S.S. (2020). Epidemiological and clinical comparisons of pediatric and adult sporotrichosis in Jilin Province, China. Mycoses.

[68] Zhang Y., Hagen F., Stielow B., Rodrigues A.M., Samerpitak K., Zhou X., Feng P., Yang L., Chen M., Deng S. (2015). Phylogeography and evolutionary patterns in *Sporothrix* spanning more than 14,000 human and animal case reports. Persoonia.

[69] Zamri-Saad M., Salmiyah T.S., Jasni S., Cheng B.Y., Basri K. (1990). Feline sporotrichosis: An increasingly important zoonotic disease in Malaysia. Vet. Rec..

[70] Tang M.M., Tang J.J., Gill P., Chang C.C., Baba R. (2012). Cutaneous sporotrichosis: A six-year review of 19 cases in a tertiary referral center in Malaysia. Int. J. Derm..

[71] Kano R., Okubo M., Siew H.H., Kamata H., Hasegawa A. (2015). Molecular typing of *Sporothrix schenckii* isolates from cats in Malaysia. Mycoses.

[72] Beurmann L., Ramond L. (1903). Abcès sous-cutanés multiples d’origine mycosique. Ann. Derm. Syph..

[73] Matruchot L. (1910). Sur un nouveau groupe de champignons pathogènes, agents des sporotrichoses. Comptes Rendus De L’Académie De Sci..

[74] Beurmann L., Gougerot H. (1912). Les Sporotrichose.

[75] Romeo O., Scordino F., Criseo G. (2011). New insight into molecular phylogeny and epidemiology of *Sporothrix schenckii* species complex based on calmodulin-encoding gene analysis of Italian isolates. Mycopathologia.

[76] Criseo G., Romeo O. (2010). Ribosomal DNA sequencing and phylogenetic analysis of environmental *Sporothrix schenckii* strains: Comparison with clinical isolates. Mycopathologia.

[77] Criseo G., Malara G., Romeo O., Puglisi Guerra A. (2008). Lymphocutaneous sporotrichosis in an immunocompetent patient: A case report from extreme southern Italy. Mycopathologia.

[78] Marimon R., Cano J., Gené J., Sutton D.A., Kawasaki M., Guarro J. (2007). *Sporothrix brasiliensis*, *S. globosa*, and *S. mexicana*, three new *Sporothrix* species of clinical interest. J. Clin. Microbiol..

[79] Madrid H., Cano J., Gene J., Bonifaz A., Toriello C., Guarro J. (2009). *Sporothrix globosa*, a pathogenic fungus with widespread geographical distribution. Rev. Iberoam. De Micol..

[80] Rachman R., Ligaj M., Chinthapalli S., Serafino Wani R. (2022). Zoonotic acquisition of cutaneous *Sporothrix brasiliensis* infection in the UK. BMJ Case Rep..

[81] McGuinness S.L., Boyd R., Kidd S., McLeod C., Krause V.L., Ralph A.P. (2016). Epidemiological investigation of an outbreak of cutaneous sporotrichosis, Northern Territory, Australia. BMC Infect. Dis..

[82] New D., Beukers A.G., Kidd S.E., Merritt A.J., Weeks K., van Hal S.J., Arthur I. (2019). Identification of multiple species and subpopulations among Australian clinical *Sporothrix* isolates using whole genome sequencing. Med. Mycol..

[83] Schauer A., Campbell A.J., Arthur I., Blyth C.C., Bowen A.C. (2020). Spotting sporotrichosis skin infection: The first Australian paediatric case series. J. Paediatr. Child Health.

[84] Mackay B., Menrath V., Ridley M., Kelly W. (1986). Sporotrichosis in a cat. Aust. Vet Pr..

[85] Thomson J., Trott D.J., Malik R., Galgut B., McAllister M.M., Nimmo J., Renton D., Kidd S.E. (2019). An atypical cause of sporotrichosis in a cat. Med. Mycol. Case Rep..

[86] Dixon D.M., Salkin I.F., Duncan R.A., Hurd N.J., Haines J.H., Kemna M.E., Coles F.B. (1991). Isolation and characterization of *Sporothrix schenckii* from clinical and environmental sources associated with the largest U.S. epidemic of sporotrichosis. J. Clin. Microbiol..

[87] Coles F.B., Schuchat A., Hibbs J.R., Kondracki S.F., Salkin I.F., Dixon D.M., Chang H.G., Duncan R.A., Hurd N.J., Morse D.L. (1992). A multistate outbreak of sporotrichosis associated with *Sphagnum* moss. Am. J. Epidemiol..

[88] Gold J.A.W., Derado G., Mody R.K., Benedict K. (2016). Sporotrichosis-associated hospitalizations, United States, 2000–2013. Emerg. Infect. Dis..

[89] Queiroz-Telles F., Fahal A.H., Falci D.R., Caceres D.H., Chiller T., Pasqualotto A.C. (2017). Neglected endemic mycoses. Lancet Infect. Dis..

[90] Hernández-Castro R., Pinto-Almazán R., Arenas R., Sánchez-Cárdenas C.D., Espinosa-Hernández V.M., Sierra-Maeda K.Y., Conde-Cuevas E., Juárez-Durán E.R., Xicohtencatl-Cortes J., Carrillo-Casas E.M. (2022). Epidemiology of clinical sporotrichosis in the Americas in the last ten years. J. Fungi.

[91] Rodrigues A.M., de Hoog G.S., Zhang Y., Camargo Z.P. (2014). Emerging sporotrichosis is driven by clonal and recombinant *Sporothrix* species. Emerg. Microbes Infect..

[92] Florez-Munoz S.V., Alzate J.F., Mesa-Arango A.C. (2019). Molecular identification and antifungal susceptibility of clinical isolates of *Sporothrix schenckii* complex in Medellin, Colombia. Mycopathologia.

[93] Ramirez Soto M.C. (2015). Sporotrichosis: The Story of an Endemic Region in Peru over 28 Years (1985 to 2012). PLoS ONE.

[94] Pappas P.G., Tellez I., Deep A.E., Nolasco D., Holgado W., Bustamante B. (2000). Sporotrichosis in Peru: Description of an area of hyperendemicity. Clin. Infect Dis..

[95] Camacho E., León-Navarro I., Rodríguez-Brito S., Mendoza M., Niño-Vega G.A. (2015). Molecular epidemiology of human sporotrichosis in Venezuela reveals high frequency of *Sporothrix globosa*. BMC Infect Dis..

[96] Rodrigues A.M., de Hoog G.S., de Camargo Z.P. (2015). Molecular diagnosis of pathogenic *Sporothrix* species. PLoS Negl. Trop. Dis..

[97] Rodrigues A.M., de Hoog G.S., de Cassia Pires D., Brilhante R.S.N., da Costa Sidrim J.J., Gadelha M.F., Colombo A.L., de Camargo Z.P. (2014). Genetic diversity and antifungal susceptibility profiles in causative agents of sporotrichosis. BMC Infect. Dis..

[98] Rodrigues A.M., de Hoog S., de Camargo Z.P. (2013). Emergence of pathogenicity in the *Sporothrix schenckii* complex. Med. Mycol..

[99] Rodrigues A.M., de Melo Teixeira M., de Hoog G.S., Schubach T.M.P., Pereira S.A., Fernandes G.F., Bezerra L.M.L., Felipe M.S., de Camargo Z.P. (2013). Phylogenetic analysis reveals a high prevalence of *Sporothrix brasiliensis* in feline sporotrichosis outbreaks. PLoS Negl. Trop. Dis..

[100] Falcão E.M.M., de Lima Filho J.B., Campos D.P., Valle A.C.F.d., Bastos F.I., Gutierrez-Galhardo M.C., Freitas D.F.S. (2019). [Hospitalizations and deaths related to sporotrichosis in Brazil (1992–2015)]. Cad. De Saude Publica.

[101] de Carvalho J.A., Beale M.A., Hagen F., Fisher M.C., Kano R., Bonifaz A., Toriello C., Negroni R., Rego R.S.M., Gremiao I.D.F. (2021). Trends in the molecular epidemiology and population genetics of emerging *Sporothrix* species. Stud. Mycol..

[102] Araujo A.K.L., de Santana Leal C.A. (2016). Feline sporotrichosis in the municipality of Bezerros, Agreste Pernambucano: Case report. Pubvet.

[103] Nunes G.D.L., dos Santos Carneiro R., Filgueira K.D., Filgueira F.G.F., Fernandes T.H.T. (2011). Feline sporotrichosis in Itaporanga municipality, Paraíba state, Brazil: Case report. Arq. De Ciências Veterinárias E Zool. Da UNIPAR.

[104] Filgueira K.D. (2009). Sporotrichosis in the canine species: A case report on city of Mossoro, RN. Cienc. Anim. Bras..

[105] de Oliveira Bento A., de Sena Costa A.S., Lima S.L., do Monte Alves M., de Azevedo Melo A.S., Rodrigues A.M., da Silva-Rocha W.P., Milan E.P., Chaves G.M. (2021). The spread of cat-transmitted sporotrichosis due to *Sporothrix brasiliensis* in Brazil towards the Northeast region. PLoS Negl. Trop. Dis..

[106] Schubach T.M., Schubach A., Okamoto T., Barros M.B., Figueiredo F.B., Cuzzi T., Fialho-Monteiro P.C., Reis R.S., Perez M.A., Wanke B. (2004). Evaluation of an epidemic of sporotrichosis in cats: 347 cases (1998–2001). J. Am. Vet. Med. Assoc..

[107] Schubach A.O., Schubach T.M., Barros M.B. (2005). Epidemic cat-transmitted sporotrichosis. N. Engl. J. Med..

[108] Falcao E.M.M., Pires M.C.S., Andrade H.B., Goncalves M.L.C., Almeida-Paes R., do Valle A.C.F., Bastos F.I., Gutierrez-Galhardo M.C., Freitas D.F.S. (2019). Zoonotic sporotrichosis with greater severity in Rio de Janeiro, Brazil: 118 hospitalizations and 11 deaths in the last 2 decades in a reference institution. Med. Mycol..

[109] Córdoba S., Isla G., Szusz W., Vivot W., Hevia A., Davel G., Canteros C.E. (2018). Molecular identification and susceptibility profile of *Sporothrix schenckii sensu lato* isolated in Argentina. Mycoses.

[110] Etchecopaz A.N., Lanza N., Toscanini M.A., Devoto T.B., Pola S.J., Daneri G.L., Iovannitti C.A., Cuestas M.L. (2019). Sporotrichosis caused by *Sporothrix brasiliensis* in Argentina: Case report, molecular identification and in vitro susceptibility pattern to antifungal drugs. J. De Mycol. Med..

[111] García Duarte J.M., Wattiez Acosta V.R., Fornerón Viera P.M.L., Aldama Caballero A., Gorostiaga Matiauda G.A., Rivelli de Oddone V.B., Pereira Brunelli J.G. (2017). Sporotrichosis transmitted by domestic cat. A family case report. Rev. Del Nac..

[112] Rios M.E., Suarez J.M.D., Moreno J., Vallee J., Moreno J.P. (2018). Zoonotic sporotrichosis related to cat contact: First case report from Panama in Central America. Cureus.

[113] PAHO *Sporothrix brasiliensis*, an Emerging Fungal Pathogen, Notable for its Zoonotic Transmission and Epidemic Potential for Human and Animal Health in the Americas. https://www.someve.com.ar/images/noticias/2019/S-brasiliensis_lasAmericas_30082019_ES.pdf.

[114] Montenegro H., Rodrigues A.M., Galvão Dias M.A., da Silva E.A., Bernardi F., Camargo Z.P. (2014). Feline sporotrichosis due to *Sporothrix brasiliensis*: An emerging animal infection in São Paulo, Brazil. BMC Vet Res..

[115] Zhou X., Rodrigues A.M., Feng P., Hoog G.S. (2014). Global ITS diversity in the *Sporothrix schenckii* complex. Fungal Divers..

[116] Rediguieri B.C., da Cruz Bahiense I., de Carvalho J.A., Leite G.R., Falqueto A., Rodrigues A.M., Gonçalves S.S. (2022). Clinical, epidemiological, and epizootic features of *Sporothrix brasiliensis* in Espírito Santo, Brazil. Ecohealth.

[117] Maschio-Lima T., Marques M.D.R., Lemes T.H., Brizzotti-Mazuchi N.S., Caetano M.H., de Almeida B.G., Bianco L.M., Monteiro R.C., Rodrigues A.M., de Camargo Z.P. (2021). Clinical and epidemiological aspects of feline sporotrichosis caused by *Sporothrix brasiliensis* and in vitro antifungal susceptibility. Vet. Res. Commun..

[118] da Cruz Bahiense Rocha I., Terra P.P.D., Cardoso de Oliveira R., Lubianca Zanotti R., Falqueto A., de Camargo Z.P., Rodrigues A.M., Goncalves S.S. (2021). Molecular-based assessment of diversity and population structure of *Sporothrix* spp. clinical isolates from Espirito Santo-Brazil. Mycoses.

[119] Macêdo-Sales P.A., Souza L.O.P., Della-Terra P.P., Lozoya-Pérez N.E., Machado R.L.D., Rocha E.M.d.S.d., Lopes-Bezerra L.M., Guimarães A.J., Rodrigues A.M., Mora-Montes H.M. (2020). Coinfection of domestic felines by distinct *Sporothrix brasiliensis* in the Brazilian sporotrichosis hyperendemic area. Fungal Genet. Biol..

[120] Macêdo-Sales P.A., Souto S.R.L.S., Destefani C.A., Lucena R.P., Machado R.L.D., Pinto M.R., Rodrigues A.M., Lopes-Bezerra L.M., Rocha E.M.S., Baptista A.R.S. (2018). Domestic feline contribution in the transmission of *Sporothrix* in Rio de Janeiro State, Brazil: A comparison between infected and non-infected populations. BMC Vet. Res..

[121] Teixeira M.d.M., Rodrigues A.M., Tsui C.K.M., de Almeida L.G.P., Van Diepeningen A.D., Gerrits van den Ende B., Fernandes G.F., Kano R., Hamelin R.C., Lopes-Bezerra L.M. (2015). Asexual propagation of a virulent clone complex in human and feline outbreak of sporotrichosis. Eukaryot Cell.

[122] Rodrigues A.M., Fernandes G.F., Araujo L.M., Della Terra P.P., Dos Santos P.O., Pereira S.A., Schubach T.M., Burger E., Lopes-Bezerra L.M., de Camargo Z.P. (2015). Proteomics-based characterization of the humoral immune response in sporotrichosis: Toward discovery of potential diagnostic and vaccine antigens. PLoS Negl. Trop. Dis..

[123] Freitas D.C., Migliano M.F., Zani Neto L. (1956). Sporotrichosis. Observation of spontaneous case in domestic cat (*Felis catus*). Rev. Fac. Med. Vet. Univ. Sao Paulo.

[124] Freitas D.C., Moreno G., Saliba A.M., Botino J.Á., Mós E.M. (1965). Sporotrichosis in dogs and cats. Rev. Fac. Med. Vet Univ. Sao Paulo.

[125] Almeida F., Sampaio S.A.P., Lacaz C.S., Fernandes J.C. (1955). Statistical data on sporotrichosis. Analysis of 344 cases. An. Bras. De Dermatol..

[126] Lacaz C.S., Porto E., Martins J.E.C., Heins-Vaccari E.M., de Melo N.T. (2002). Tratado de Micologia Médica.

[127] Marques S.A., Franco S.R., de Camargo R.M., Dias L.D., Haddad Junior V., Fabris V.E. (1993). Sporotrichosis of the domestic cat (*Felis catus*): Human transmission. Rev. Do Inst. De Med. Trop. De Sao Paulo.

[128] Baroni F., Campos S., Direito G. (1998). Sporotrichosis in a cat (a case description). Rev. Bras. Med. Vet..

[129] Barros M.B.d.L., Schubach T.P., Coll J.O., Gremião I.D., Wanke B., Schubach A. (2010). Sporotrichosis: Development and challenges of an epidemic. Rev. Panam. Salud Publica.

[130] Gremião I.D.F., Oliveira M.M.E., Monteiro de Miranda L.H., Saraiva Freitas D.F., Pereira S.A. (2020). Geographic expansion of sporotrichosis, Brazil. Emerg. Infect. Dis..

[131] Larsson C.E., Goncalves Mde A., Araujo V.C., Dagli M.L., Correa B., Fava Neto C. (1989). Feline sporotrichosis: Clinical and zoonotic aspects. Rev. Do Inst. De Med. Trop. De Sao Paulo.

[132] Borges T.S., Rossi C.N., Fedullo J.D., Taborda C.P., Larsson C.E. (2013). Isolation of *Sporothrix schenckii* from the claws of domestic cats (indoor and outdoor) and in captivity in Sao Paulo (Brazil). Mycopathologia.

[133] Silva C.E., Valeriano C.A.T., Ferraz C.E., Neves R.P., Oliveira M.M.E., Silva J.C.A.L., Magalhães V., Lima-Neto R.G. (2021). Epidemiological features and geographical expansion of sporotrichosis in the state of Pernambuco, northeastern Brazil. Future Microbiol..

[134] Larsson C.E. (2011). Sporotrichosis. Braz. J. Vet. Res. Anim. Sci..

[135] Almeida F.P. (1939). Micologia Médica: Estudo das Micoses Humanas e de Seus Cogumelos.

[136] Fernandes C.G.N., Moura S.T.d., Dantas A.F.M., Blatt M.C.S. (2004). Feline sporotrichosis: Clinical epidemiological aspects: Case reports (Cuiabá, Mato Grosso, Brasil). MEDVEP. Rev. Cient. Med. Vet..

[137] SES-MS Sporotrichosis Technical Note—Nº 1/2021-GTZ/CEVE/DGVS/SES. https://www.vs.saude.ms.gov.br/wp-content/uploads/2021/08/Nota-tecnica-esporotricose.pdf.

[138] CRMV-SE Technical Note No. 01/2021. https://www.crmvse.org.br/wp-content/uploads/2021/06/Nota-Tecnica-01-2021.pdf.

[139] SES-AM Joint Technical Note—Nº 006/2021—GEVEP/DEVAE/DAP/DRA/SUBGS. https://semsa.manaus.am.gov.br/wp-content/uploads/2021/05/Nota-Tecnica_Fluxo_APS_e_Vigilancia_e_Ficha_de_Notificacao_da_Esporotricose_assinadas.pdf.

[140] COVISA Technical Note 09: DVE/DVZ/COVISA/2020—Surveillance and Clinical Management of Human Sporotrichosis in the Municipality of São Paulo. https://docs.bvsalud.org/biblioref/2020/08/1102196/nota-tecnica-09-dve-zoo-2020_esporotricose_v6-alterada-a-pedid_CBJA7E3.pdf.

[141] Bison I. (2019). Feline Sporotrichosis: Literature Review. Bachelor’s Thesis.

[142] Boechat J.S., Oliveira M.M.E., Almeida-Paes R., Gremiao I.D.F., Machado A.C.S., Oliveira R.V.C., Figueiredo A.B.F., Rabello V.B.S., Silva K.B.L., Zancope-Oliveira R.M. (2018). Feline sporotrichosis: Associations between clinical-epidemiological profiles and phenotypic-genotypic characteristics of the etiological agents in the Rio de Janeiro epizootic area. Mem. Do Inst. Oswaldo Cruz.

[143] de Souza E.W., Borba C.M., Pereira S.A., Gremiao I.D.F., Langohr I.M., Oliveira M.M.E., de Oliveira R.V.C., da Cunha C.R., Zancope-Oliveira R.M., de Miranda L.H.M. (2018). Clinical features, fungal load, coinfections, histological skin changes, and itraconazole treatment response of cats with sporotrichosis caused by *Sporothrix brasiliensis*. Sci. Rep..

[144] de Araujo M.L., Rodrigues A.M., Fernandes G.F., de Camargo Z.P., de Hoog G.S. (2015). Human sporotrichosis beyond the epidemic front reveals classical transmission types in Espírito Santo, Brazil. Mycoses.

[145] Colodel M.M., Jark P.C., Ramos C.J.R., Martins V.M.V., Schneider A.F., Pilati C. (2009). Feline cutaneous sporotrichosis in the state of Santa Catarina: Case reports. Vet. Foco.

[146] Cordeiro F.N., Bruno C.B., Paula C.D., Motta Jde O. (2011). Familial occurrence of zoonotic sporotrichosis. An. Bras. De Dermatol..

[147] Silva G.M., Howes J.C.F., Leal C.A.S., Mesquita E.P., Pedrosa C.M., Oliveira A.A.F., Silva L.B.G., Mota R.A. (2018). Feline sporotrichosis outbreak in metropolitan Recife. Pesqui. Vet. Bras..

[148] Marques-Melo E.H., Lessa D.F.d.S., Garrido L.H.A., Nunes A.C.B.T., Chaves K.P., Porto W.J.N., Notomi M. (2014). Domestic feline as sporotricosis transmitting agent for human: Report of the first case in the state of Alagoas. Rev. Baiana De Saúde Pública.

[149] Madrid I.M., Mattei A., Martins A., Nobre M., Meireles M. (2010). Feline sporotrichosis in the southern region of Rio Grande do Sul, Brazil: Clinical, zoonotic and therapeutic aspects. Zoonoses Public Health.

[150] Sanchotene K.O., Madrid I.M., Klafke G.B., Bergamashi M., Terra P.P.D., Rodrigues A.M., de Camargo Z.P., Xavier M.O. (2015). *Sporothrix brasiliensis* outbreaks and the rapid emergence of feline sporotrichosis. Mycoses.

[151] Madrid I.M., Mattei A.S., Fernandes C.G., Oliveira Nobre M., Meireles M.C.A. (2012). Epidemiological findings and laboratory evaluation of sporotrichosis: A description of 103 cases in cats and dogs in Southern Brazil. Mycopathologia.

[152] Eudes Filho J., Santos I.B.D., Reis C.M.S., Patané J.S.L., Paredes V., Bernardes J., Poggiani S., Castro T.C.B., Gomez O.M., Pereira S.A. (2020). A novel *Sporothrix brasiliensis* genomic variant in Midwestern Brazil: Evidence for an older and wider sporotrichosis epidemic. Emerg. Microbes Infect..

[153] Bernardes-Engemann A.R., Almeida M.A., Bison I., Rabello V.B.S., Ramos M.L.M., Pereira S.A., Almeida-Paes R., de Lima Brasil A.W., Zancopé-Oliveira R.M. (2022). Anti-*Sporothrix* Antibody Detection in Domestic Cats as an Indicator of a Possible New Occurrence Area for Sporotrichosis in North Brazil. Mycopathologia.

[154] Schmidt P.M., Lopez R.R., Collier B.A. (2007). Survival, fecundity, and movements of free-roaming cats. J. Wildl. Manag..

[155] Jongman E.C. (2007). Adaptation of domestic cats to confinement. J. Vet. Behav..

[156] Horn J.A., Mateus-Pinilla N., Warner R.E., Heske E.J. (2011). Home range, habitat use, and activity patterns of free-roaming domestic cats. J. Wildl. Manag..

[157] Keesing F., Belden L.K., Daszak P., Dobson A., Harvell C.D., Holt R.D., Hudson P., Jolles A., Jones K.E., Mitchell C.E. (2010). Impacts of biodiversity on the emergence and transmission of infectious diseases. Nature.

[158] Jones K.E., Patel N.G., Levy M.A., Storeygard A., Balk D., Gittleman J.L., Daszak P. (2008). Global trends in emerging infectious diseases. Nature.

[159] Shaheen M.N.F. (2022). The concept of one health applied to the problem of zoonotic diseases. Rev. Med. Virol..

[160] Winck G.R., Raimundo R.L.G., Fernandes-Ferreira H., Bueno M.G., D’Andrea P.S., Rocha F.L., Cruz G.L.T., Vilar E.M., Brandão M., Cordeiro J.L.P. (2022). Socioecological vulnerability and the risk of zoonotic disease emergence in Brazil. Sci. Adv..

[161] Bloom D.E., Cadarette D. (2019). Infectious disease threats in the twenty-first century: Strengthening the global response. Front. Immunol..

[162] Alzuguir C.L.C., Pereira S.A., Magalhães M.A.F.M., Almeida-Paes R., Freitas D.F.S., Oliveira L.F.A., Pimentel M.I.F. (2020). Geo-epidemiology and socioeconomic aspects of human sporotrichosis in the municipality of Duque de Caxias, Rio de Janeiro, Brazil, between 2007 and 2016. Trans. R. Soc. Trop. Med. Hyg..

[163] Silva M.B., Costa M.M., Torres C.C., Galhardo M.C., Valle A.C., Magalhaes Mde A., Sabroza P.C., Oliveira R.M. (2012). [Urban sporotrichosis: A neglected epidemic in Rio de Janeiro, Brazil]. Cad. De Saude Publica.

[164] Veasey J.V., Carvalho G.d.S.M., Ruiz L.R.B., Neves Neto M.F., Zaitz C. (2022). Epidemiological and geographical distribution profile of urban sporotrichosis in the city of São Paulo. An. Bras. De Dermatol..

[165] Ellwanger J.H., Kulmann-Leal B., Kaminski V.L., Valverde-Villegas J.M., Veiga A., Spilki F.R., Fearnside P.M., Caesar L., Giatti L.L., Wallau G.L. (2020). Beyond diversity loss and climate change: Impacts of Amazon deforestation on infectious diseases and public health. An. Da Acad. Bras. De Cienc..

[166] Pörtner H.-O., Roberts D.C., Adams H., Adelekan I., Adler C., Adrian R., Aldunce P., Ali E., Ara Begum R., Bednar-Friedl B., Pörtner H.-O., Roberts D.C., Tignor M., Poloczanska E.S., Mintenbeck K., Alegría A., Craig M., Langsdorf S., Löschke S., Möller V. (2022). IPCC, 2022: Climate Change 2022: Impacts, Adaptation, and Vulnerability: Contribution of Working Group II to the Sixth Assessment Report of the Intergovernmental Panel on Climate Change.

[167] Jones Bryony A., Grace D., Kock R., Alonso S., Rushton J., Said Mohammed Y., McKeever D., Mutua F., Young J., McDermott J. (2013). Zoonosis emergence linked to agricultural intensification and environmental change. Proc. Natl. Acad. Sci. USA.

[168] Rabello V.B.S., Almeida-Silva F., Scramignon-Costa B.d.S., Motta B.d.S., de Macedo P.M., Teixeira M.d.M., Almeida-Paes R., Irinyi L., Meyer W., Zancopé-Oliveira R.M. (2022). Environmental isolation of *Sporothrix brasiliensis* in an area with recurrent feline sporotrichosis cases. Front. Cell. Infect. Microbiol..

[169] Almeida-Silva F., Rabello V.B., Scramignon-Costa B.D., Zancopé-Oliveira R.M., de Macedo P.M., Almeida-Paes R. (2022). Beyond domestic cats: Environmental detection of *Sporothrix brasiliensis* DNA in a hyperendemic area of sporotrichosis in Rio de Janeiro state, Brazil. J. Fungi.

[170] Navarrete A.A., Tsai S.M., Mendes L.W., Faust K., de Hollander M., Cassman N.A., Raes J., van Veen J.A., Kuramae E.E. (2015). Soil microbiome responses to the short-term effects of Amazonian deforestation. Mol. Ecol..

[171] Khan M.A.W., Bohannan B.J.M., Nüsslein K., Tiedje J.M., Tringe S.G., Parlade E., Barberán A., Rodrigues J.L.M. (2019). Deforestation impacts network co-occurrence patterns of microbial communities in Amazon soils. FEMS Microbiol. Ecol..

[172] Rosenberg K., Bertaux J., Krome K., Hartmann A., Scheu S., Bonkowski M. (2009). Soil amoebae rapidly change bacterial community composition in the rhizosphere of *Arabidopsis thaliana*. ISME J..

[173] Steenbergen J.N., Nosanchuk J.D., Malliaris S.D., Casadevall A. (2004). Interaction of *Blastomyces dermatitidis*, *Sporothrix schenckii*, and *Histoplasma capsulatum* with *Acanthamoeba castellanii*. Infect. Immun..

[174] Lemos Tavares P., Carvalho Ribeiro A., Kercher Berte F., da Silva Hellwig A.H., Machado Pagani D., Tavares de Souza C.C., Brittes Rott M., Scroferneker M.L. (2020). The interaction between *Sporothrix schenckii sensu stricto* and *Sporothrix brasiliensis* with *Acanthamoeba castellanii*. Mycoses.

[175] Aqeel Y., Siddiqui R., Iftikhar H., Khan N.A. (2013). The effect of different environmental conditions on the encystation of *Acanthamoeba castellanii* belonging to the T4 genotype. Exp. Parasitol..

[176] Rossow J.A., Queiroz-Telles F., Caceres D.H., Beer K.D., Jackson B.R., Pereira J.G., Ferreira Gremião I.D., Pereira S.A. (2020). A one health approach to combatting *Sporothrix brasiliensis*: Narrative review of an emerging zoonotic fungal pathogen in South America. J. Fungi.

[177] Mahajan V.K. (2014). Sporotrichosis: An overview and therapeutic options. Dermatol. Res. Pract..

[178] Lopes-Bezerra L.M., Mora-Montes H.M., Zhang Y., Nino-Vega G., Rodrigues A.M., de Camargo Z.P., de Hoog S. (2018). Sporotrichosis between 1898 and 2017: The evolution of knowledge on a changeable disease and on emerging etiological agents. Med. Mycol..

[179] Kwon-Chung J.K., Bennett J.E. (1992). Medical Mycology.

[180] Rippon J.W. (1988). Medical Mycology—The Pathogenic Fungi and the Pathogenic Actinomycetes.

[181] Rodrigues A.M., Orofino-Costa R., de Camargo Z.P., Cordeiro Rde A. (2019). *Sporothrix* spp.. Pocket Guide to Mycological Diagnosis.

[182] Rodrigues A.M., de Hoog G.S., de Camargo Z.P., Seyedmousavi S., de Hoog G.S., Guillot J., Verweij P.E. (2018). Feline Sporotrichosis. Emerging and Epizootic Fungal Infections in Animals.

[183] Miranda L.H., Quintella L.P., Menezes R.C., dos Santos I.B., Oliveira R.V., Figueiredo F.B., Lopes-Bezerra L.M., Schubach T.M. (2011). Evaluation of immunohistochemistry for the diagnosis of sporotrichosis in dogs. Vet. J..

[184] Bernardes-Engemann A.R., de Lima Barros M., Zeitune T., Russi D.C., Orofino-Costa R., Lopes-Bezerra L.M. (2015). Validation of a serodiagnostic test for sporotrichosis: A follow-up study of patients related to the Rio de Janeiro zoonotic outbreak. Med. Mycol..

[185] Gezuele E., Da Rosa D. (2005). Importance of the sporotrichosis asteroid body for the rapid diagnosis of sporotrichosis. Rev. Iberoam. De Micol..

[186] Schwarz J. (1982). The diagnosis of deep mycoses by morphologic methods. Hum. Pathol..

[187] Morris-Jones R. (2002). Sporotrichosis. Clin. Exp. Dermatol..

[188] de Lima Barros M.B., Schubach A.O., de Vasconcellos Carvalhaes de Oliveira R., Martins E.B., Teixeira J.L., Wanke B. (2011). Treatment of cutaneous sporotrichosis with Itraconazole—Study of 645 patients. Clin. Infect. Dis..

[189] Rodrigues A.M., Hagen F., de Camargo Z.P. (2022). A spotlight on *Sporothrix* and sporotrichosis. Mycopathologia.

[190] Gonçalves S.S., Cano J.F., Stchigel A.M., Melo A.S., Godoy-Martinez P.C., Correa B., Guarro J. (2012). Molecular phylogeny and phenotypic variability of clinical and environmental strains of *Aspergillus flavus*. Fungal Biol..

[191] Silva J.N., Miranda L.H.M., Menezes R.C., Gremiao I.D.F., Oliveira R.V.C., Vieira S.M.M., Conceicao-Silva F., Ferreiro L., Pereira S.A. (2018). Comparison of the sensitivity of three methods for the early diagnosis of sporotrichosis in cats. J. Comp. Pathol..

[192] Rodrigues A.M., Fernandes G.F., de Camargo Z.P., Bayry J. (2017). Sporotrichosis. Emerging and Re-Emerging Infectious Diseases of Livestock.

[193] Marimon R., Gené J., Cano J., Guarro J. (2008). *Sporothrix luriei*: A rare fungus from clinical origin. Med. Mycol..

[194] Oliveira M.M., Almeida-Paes R., Muniz M.M., Gutierrez-Galhardo M.C., Zancope-Oliveira R.M. (2011). Phenotypic and molecular identification of *Sporothrix* isolates from an epidemic area of sporotrichosis in Brazil. Mycopathologia.

[195] Zhao M.-d., Zhou X., Liu T.-t., Yang Z.-b. (2015). Morphological and physiological comparison of taxa comprising the Sporothrix schenckii complex. J. Zhejiang Univ. Sci. B.

[196] Vásquez-del-Mercado E., Arenas R., Padilla-Desgarenes C. (2012). Sporotrichosis. Clin. Derm..

[197] Richardson M.D., Warnock D.W. (2012). Fungal Infection: Diagnosis and Management.

[198] Oyarce J.A., García C., Alave J., Bustamante B. (2016). Epidemiological clinical and laboratory characterization of sporotrichosis in patients of a tertiary care hospital in Lima, Peru, from 1991 to 2014. Rev. Chil. Infectol..

[199] Arenas R., Sanchez-Cardenas C.D., Ramirez-Hobak L., Ruiz Arriaga L.F., Vega Memije M.E. (2018). Sporotrichosis: From KOH to molecular biology. J. Fungi.

[200] Quintella L.P., Passos S.R., do Vale A.C., Galhardo M.C., Barros M.B., Cuzzi T., Reis Rdos S., de Carvalho M.H., Zappa M.B., Schubach Ade O. (2011). Histopathology of cutaneous sporotrichosis in Rio de Janeiro: A series of 119 consecutive cases. J. Cutan. Pathol..

[201] Quintella L.P., Passos S.R., de Miranda L.H., Cuzzi T., Barros M.B., Francesconi-do-Vale A.C., Galhardo M.C., Madeira Mde F., Figueiredo de Carvalho M.H., Schubach Ade O. (2012). Proposal of a histopathological predictive rule for the differential diagnosis between American tegumentary leishmaniasis and sporotrichosis skin lesions. Br. J. Dermatol..

[202] da Rosa W.D., Gezuele E., Calegari L., Goni F. (2008). Asteroid body in sporotrichosis. Yeast viability and biological significance within the host immune response. Med. Mycol..

[203] Civila E.S., Bonasse J., Conti-Diaz I.A., Vignale R.A. (2004). Importance of the direct fresh examination in the diagnosis of cutaneous sporotrichosis. Int. J. Derm..

[204] Miranda L.H., Conceicao-Silva F., Quintella L.P., Kuraiem B.P., Pereira S.A., Schubach T.M. (2013). Feline sporotrichosis: Histopathological profile of cutaneous lesions and their correlation with clinical presentation. Comp. Immunol. Microbiol. Infect. Dis..

[205] Welsh R.D. (2003). Sporotrichosis. J. Am. Vet. Med. Assoc..

[206] Pereira S.A., Menezes R.C., Gremião I.D.F., Silva J.N., de O. Honse C., Figueiredo C., da Silva D.T., Kitada A.A.B., dos Reis É.G., Schubach T.M.P. (2011). Sensitivity of cytopathological examination in the diagnosis of feline sporotrichosis. J. Feline Med. Surg..

[207] Seyedmousavi S., Bosco S.d.M.G., de Hoog S., Ebel F., Elad D., Gomes R.R., Jacobsen I.D., Jensen H.E., Martel A., Mignon B. (2018). Fungal infections in animals: A patchwork of different situations. Med. Mycol..

[208] Gonsales F.F., Fernandes N., Mansho W., Montenegro H., Guerra J.M., de Araujo L.J.T., da Silva S.M.P., Benites N.R. (2019). Feline *Sporothrix* spp. detection using cell blocks from brushings and fine-needle aspirates: Performance and comparisons with culture and histopathology. Vet. Clin. Pathol..

[209] Zhang Y.Q., Xu X.G., Zhang M., Jiang P., Zhou X.Y., Li Z.Z., Zhang M.F. (2011). Sporotrichosis: Clinical and histopathological manifestations. Am. J. Dermatopathol..

[210] Hussein M.R. (2008). Mucocutaneous Splendore-Hoeppli phenomenon. J. Cutan. Pathol..

[211] Widal F., Aerami P., Joltrain E., Brissaud E.T., Weill A. (1910). Serodiagnostic mycosique, applications au diagnostic de la sporotrichose et de l’actinomycose. Les coagulations et cofixations mycosiques. Ann. Inst. Pasteur..

[212] Ochoa A.G., Figueroa E. (1947). Polisacaridos del *Sporotrichum schenckii*. Datos immunologicos: Intradermoreaccion en el diagnostico de la esporotrichosis. Rev. Inst. Salubr. Enferm. Trop..

[213] Blumer S.O., Kaufman L., Kaplan W., McLaughlin D.W., Kraft D.E. (1973). Comparative evaluation of five serological methods for the diagnosis of sporotrichosis. Appl. Microbiol..

[214] Velasco O., Ochoa A.G. (1971). Sporotrichosis in patients with previous positive sporotrichin reaction. Rev. De Investig. En Salud Publica.

[215] Ghosh A., Chakrabarti A., Sharma V.K., Singh K., Singh A. (1999). Sporotrichosis in Himachal Pradesh (north India). Trans. R. Soc. Trop. Med. Hyg..

[216] Bonifaz A., Vázquez-González D. (2010). Sporotrichosis: An update. G Ital Derm. Venereol.

[217] Kusuhara M. (2009). Sporotrichosis and dematiaceous fungal skin infections. Nihon Ishinkin Gakkai Zasshi.

[218] Itoh M., Okamoto S., Kariya H. (1986). Survey of 200 cases of sporotrichosis. Dermatologica.

[219] Kusuhara M., Hachisuka H., Sasai Y. (1988). Statistical survey of 150 cases with sporotrichosis. Mycopathologia.

[220] Posada H.R. (1968). Prueba cutanea con esporotricina. Mycopathologia.

[221] Bonifaz A., Toriello C., Araiza J., Ramírez-Soto M.C., Tirado-Sánchez A. (2018). Sporotrichin skin test for the diagnosis of sporotrichosis. J. Fungi.

[222] Bernardes-Engemann A.R., Orofino Costa R.C., Miguens B.P., Penha C.V.L., Neves E., Pereira B.A.S., Dias C.M.P., Mattos M., Gutierrez M.C., Schubach A. (2005). Development of an enzyme-linked immunosorbent assay for the serodiagnosis of several clinical forms of sporotrichosis. Med. Mycol..

[223] López-Ribot J.L., Casanova M., Murgui A., Martínez J.P. (2004). Antibody response to *Candida albicans* cell wall antigens. FEMS Immunol. Med. Microbiol..

[224] Alba-Fierro C.A., Pérez-Torres A., López-Romero E., Cuéllar-Cruz M., Ruiz-Baca E. (2014). Cell wall proteins of *Sporothrix schenckii* as immunoprotective agents. Rev. Iberoam. De Micol..

[225] Fernandes G.F., Lopes-Bezerra L.M., Bernardes-Engemann A.R., Schubach T.M., Dias M.A., Pereira S.A., de Camargo Z.P. (2011). Serodiagnosis of sporotrichosis infection in cats by enzyme-linked immunosorbent assay using a specific antigen, SsCBF, and crude exoantigens. Vet. Microbiol..

[226] Penha C.V., Bezerra L.M. (2000). Concanavalin A-binding cell wall antigens of *Sporothrix schenckii*: A serological study. Med. Mycol..

[227] Alvarado P., Ostos A., Franquiz N., Roschman-González A., Zambrano E.A., Mendoza M. (2015). Serological diagnosis of sporotrichosis using an antigen of *Sporothrix schenckii sensu stricto* mycelium. Investig. Clin..

[228] Fernandes G.F., Amaral C.C.D., Sasaki A., Godoy P.M., De Camargo Z.P. (2009). Heterogeneity of proteins expressed by Brazilian *Sporothrix schenckii* isolates. Med. Mycol..

[229] Bernardes-Engemann A.R., Loureiro y Penha C.V., Benvenuto F., Braga J.U., Barros M.L., Orofino-Costa R., Lopes-Bezerra L.M. (2009). A comparative serological study of the SsCBF antigenic fraction isolated from three *Sporothrix schenckii* strains. Med. Mycol..

[230] Rodrigues A.M., Kubitschek-Barreira P.H., Fernandes G.F., de Almeida S.R., Lopes-Bezerra L.M., de Camargo Z.P. (2015). Immunoproteomic analysis reveals a convergent humoral response signature in the *Sporothrix schenckii* complex. J. Proteom..

[231] Martínez-Álvarez J.A., García-Carnero L.C., Kubitschek-Barreira P.H., Lozoya-Pérez N.E., Belmonte-Vázquez J.L., de Almeida J.R., Gómez-Infante A.D.J., Curty N., Villagómez-Castro J.C., Peña-Cabrera E. (2019). Analysis of some immunogenic properties of the recombinant *Sporothrix schenckii* Gp70 expressed in *Escherichia coli*. Future Microbiol..

[232] Baptista V.S., Mothé G.B., Santos G.M.P., Melivilu C.S.I., Santos T.O., Virginio E.D., de Macêdo-Sales P.A., Pinto M.R., Machado R.L.D., Rocha E.M.S. (2021). Promising application of the SsCBF ELISA test to monitor the therapeutic response of feline sporotrichosis caused by *Sporothrix brasiliensis* from Brazilian epidemics. Braz. J. Microbiol..

[233] Elias Costa M.R., Da Silva Lacaz C., Kawasaki M., De Camargo Z.P. (2000). Conventional versus molecular diagnostic tests. Med. Mycol..

[234] Ghosh A., Maity P.K., Hemashettar B.M., Sharma V.K., Chakrabarti A. (2002). Physiological characters of *Sporothrix schenckii* isolates. Mycoses.

[235] Mesa-Arango A.C., del Rocío Reyes-Montes M., Pérez-Mejía A., Navarro-Barranco H., Souza V., Zúñiga G., Toriello C. (2002). Phenotyping and genotyping of *Sporothrix schenckii* isolates according to geographic origin and clinical form of sporotrichosis. J. Clin. Microbiol..

[236] Lücking R., Aime M.C., Robbertse B., Miller A.N., Aoki T., Ariyawansa H.A., Cardinali G., Crous P.W., Druzhinina I.S., Geiser D.M. (2021). Fungal taxonomy and sequence-based nomenclature. Nat. Microbiol..

[237] Crous P.W., Lombard L., Sandoval-Denis M., Seifert K.A., Schroers H.J., Chaverri P., Gené J., Guarro J., Hirooka Y., Bensch K. (2021). *Fusarium*: More than a node or a foot-shaped basal cell. Stud. Mycol..

[238] Della Terra P.P., Gonsales F.F., de Carvalho J.A., Hagen F., Kano R., Bonifaz A., Camargo Z.P., Rodrigues A.M. (2021). Development and evaluation of a multiplex qPCR assay for rapid diagnostics of emerging sporotrichosis. Transbound. Emerg. Dis..

[239] Pinheiro B.G., Hahn R.C., Camargo Z.P., Rodrigues A.M. (2020). Molecular tools for detection and identification of *Paracoccidioides* species: Current status and future perspectives. J. Fungi.

[240] de Carvalho J.A., Monteiro R.C., Hagen F., Camargo Z.P., Rodrigues A.M. (2022). Trends in molecular diagnostics and genotyping tools applied for emerging *Sporothrix* species. J. Fungi.

[241] Hu S., Chung W.-H., Hung S.-I., Ho H.-C., Wang Z.-W., Chen C.-H., Lu S.-C., Kuo T.-T., Hong H.-S. (2003). Detection of *Sporothrix schenckii* in clinical samples by a nested PCR assay. J. Clin. Microbiol..

[242] Kano R., Nakamura Y., Watanabe S., Tsujimoto H., Hasegawa A. (2001). Identification of *Sporothrix schenckii* based on sequences of the chitin synthase 1 gene. Mycoses.

[243] Kano R., Matsuoka A., Kashima M., Nakamura Y., Watanabe S., Mizoguchi M., Hasegawa A. (2003). Detection of *Sporothrix schenckii* chitin synthase 1 (CHS1) gene in biopsy specimens from human patients with sporotrichosis. J. Derm. Sci..

[244] Mendoza M., Brito A., Schaper D.A., Spooner V.A., Alvarado P., Castro A., Fernandez A. (2012). Technical evaluation of nested PCR for the diagnosis of experimental sporotrichosis. Rev. Iberoam. De Micol..

[245] Zhang M., Li F., Gong J., Yang X., Zhang J., Zhao F. (2019). Development and evaluation of a real-time polymerase chain reaction for fast diagnosis of sporotrichosis caused by *Sporothrix globosa*. Med. Mycol..

[246] Rodrigues A.M., de Hoog G.S., Camargo Z.P. (2014). Genotyping species of the *Sporothrix schenckii* complex by PCR-RFLP of calmodulin. Diagn Microbiol. Infect. Dis..

[247] Rodrigues A.M., Najafzadeh M.J., de Hoog G.S., de Camargo Z.P. (2015). Rapid identification of emerging human-pathogenic *Sporothrix* species with rolling circle amplification. Front. Microbiol..

[248] Ramírez-Soto M.C., Aguilar-Ancori E.G., Quispe-Ricalde M.A., Muñiz-Duran J.G., Quispe-Florez M.M., Chinen A. (2021). Molecular identification of *Sporothrix* species in a hyperendemic area in Peru. J. Infect. Public. Health..

[249] Gonsales F.F., Fernandes N.C.C.A., Mansho W., Montenegro H., Benites N.R. (2020). Direct PCR of lesions suggestive of sporotrichosis in felines. Arq. Bras. Med. Vet. Zootec..

[250] Zhang M., Li F., Li R., Gong J., Zhao F. (2019). Fast diagnosis of sporotrichosis caused by *Sporothrix globosa*, *Sporothrix schenckii*, and *Sporothrix brasiliensis* based on multiplex real-time PCR. PLoS Negl. Trop. Dis..

[251] Madrid H., Gené J., Cano J., Silvera C., Guarro J. (2010). *Sporothrix brunneoviolacea* and *Sporothrix dimorphospora*, two new members of the *Ophiostoma stenoceras*-*Sporothrix schenckii* complex. Mycologia.

[252] Rangel-Gamboa L., Martinez-Hernandez F., Maravilla P., Arenas-Guzman R., Flisser A. (2016). Update of phylogenetic and genetic diversity of *Sporothrix schenckii sensu lato*. Med. Mycol..

[253] de Carvalho J.A., Hagen F., Fisher M.C., de Camargo Z.P., Rodrigues A.M. (2020). Genome-wide mapping using new AFLP markers to explore intraspecific variation among pathogenic *Sporothrix* species. PLoS Negl. Trop. Dis..

[254] Restrepo C.M., Llanes A., Lleonart R. (2018). Use of AFLP for the study of eukaryotic pathogens affecting humans. Infect. Genet. Evol..

[255] Roberto T.N., De Carvalho J.A., Beale M.A., Hagen F., Fisher M.C., Hahn R.C., de Camargo Z.P., Rodrigues A.M. (2021). Exploring genetic diversity, population structure, and phylogeography in *Paracoccidioides* species using AFLP markers. Stud. Mycol..

[256] Rodrigues A.M., Beale M.A., Hagen F., Fisher M.C., Terra P.P.D., de Hoog S., Brilhante R.S.N., de Aguiar Cordeiro R., de Souza Collares Maia Castelo-Branco D., Rocha M.F.G. (2020). The global epidemiology of emerging *Histoplasma* species in recent years. Stud. Mycol..

[257] Singhal N., Kumar M., Kanaujia P.K., Virdi J.S. (2015). MALDI-TOF mass spectrometry: An emerging technology for microbial identification and diagnosis. Front. Microbiol..

[258] Murray P.R. (2012). What is new in clinical microbiology-microbial identification by MALDI-TOF mass spectrometry: A paper from the 2011 William Beaumont Hospital Symposium on molecular pathology. J. Mol. Diagn. JMD.

[259] Oliveira M.M., Santos C., Sampaio P., Romeo O., Almeida-Paes R., Pais C., Lima N., Zancope-Oliveira R.M. (2015). Development and optimization of a new MALDI-TOF protocol for identification of the *Sporothrix* species complex. Res. Microbiol..

[260] Etchecopaz A., Toscanini M.A., Gisbert A., Mas J., Scarpa M., Iovannitti C.A., Bendezú K., Nusblat A.D., Iachini R., Cuestas M.L. (2021). Sporothrix brasiliensis: A review of an emerging South American fungal pathogen, its related disease, presentation and spread in Argentina. J. Fungi.

[261] Matos A.M.F., Moreira L.M., Barczewski B.F., de Matos L.X., de Oliveira J.B.V., Pimentel M.I.F., Almeida-Paes R., Oliveira M.G., Pinto T.C.A., Lima N. (2019). Identification by MALDI-TOF MS of *Sporothrix brasiliensis* isolated from a subconjunctival infiltrative lesion in an immunocompetent patient. Microorganisms.

[262] Espinel-Ingroff A., Abreu D.P.B., Almeida-Paes R., Brilhante R.S.N., Chakrabarti A., Chowdhary A., Hagen F., Cordoba S., Gonzalez G.M., Govender N.P. (2017). Multicenter and international study of MIC/MEC distributions for definition of epidemiological cutoff values (ECVs) for species of *Sporothrix* identified by molecular methods. Antimicrob. Agents Chemother..

[263] Costa R.O., Macedo P.M., Carvalhal A., Bernardes-Engemann A.R. (2013). Use of potassium iodide in dermatology: Updates on an old drug. An. Bras. De Dermatol..

[264] Brilhante R.S.N., Silva M., Pereira V.S., de Oliveira J.S., Maciel J.M., Silva I., Garcia L.G.S., Guedes G.M.M., Cordeiro R.A., Pereira-Neto W.A. (2018). Potassium iodide and miltefosine inhibit biofilms of *Sporothrix schenckii* species complex in yeast and filamentous forms. Med. Mycol..

[265] Sharma B., Sharma A.K., Sharma U. (2022). Sporotrichosis: A comprehensive review on recent drug-based therapeutics and management. Curr. Derm. Rep..

[266] Gremião I.D.F., Miranda L.H.M.d., Pereira-Oliveira G.R., Menezes R.C., Machado A.C.d.S., Rodrigues A.M., Pereira S.A. (2022). Advances and challenges in the management of feline sporotrichosis. Rev. Iberoam. Micol..

[267] Borba-Santos L.P., Rodrigues A.M., Gagini T.B., Fernandes G.F., Castro R., de Camargo Z.P., Nucci M., Lopes-Bezerra L.M., Ishida K., Rozental S. (2015). Susceptibility of *Sporothrix brasiliensis* isolates to amphotericin B, azoles, and terbinafine. Med. Mycol..

[268] CDDI Cortellis Drug Discovery Intelligence Database. https://www.cortellis.com/drugdiscovery/.

[269] Francesconi G., Francesconi do Valle A.C., Passos S.L., de Lima Barros M.B., de Almeida Paes R., Curi A.L., Liporage J., Porto C.F., Galhardo M.C. (2011). Comparative study of 250 mg/day terbinafine and 100 mg/day itraconazole for the treatment of cutaneous sporotrichosis. Mycopathologia.

[270] Carolus H., Pierson S., Lagrou K., Van Dijck P. (2020). Amphotericin B and other polyenes-discovery, clinical use, mode of action and drug resistance. J. Fungi.

[271] Institute C.a.L.S. (2017). Reference Method for Broth Dilution Antifungal Susceptibility Testing of Yeast, M27.

[272] Institute C.a.L.S. (2017). Reference Method for Broth Dilution Antifungal Susceptibility Testing of Filamentous Fungi, Approved Standard, M38.

[273] Fernandez-Silva F., Capilla J., Mayayo E., Guarro J. (2014). Modest efficacy of voriconazole against murine infections by *Sporothrix schenckii* and lack of efficacy against *Sporothrix brasiliensis*. Mycoses.

[274] Mario D.N., Guarro J., Santurio J.M., Alves S.H., Capilla J. (2015). In vitro and in vivo efficacy of amphotericin B combined with posaconazole against experimental disseminated sporotrichosis. Antimicrob. Agents Chemother..

[275] Fichman V., Valle A.C.F.d., de Macedo P.M., Freitas D.F.S., Oliveira M.M.E.d., Almeida-Paes R., Gutierrez-Galhardo M.C. (2018). Cryosurgery for the treatment of cutaneous sporotrichosis in four pregnant women. PLoS Negl. Trop. Dis..

[276] Ferreira C.P., do Valle A.C., Freitas D.F., Reis R., Galhardo M.C. (2012). Pregnancy during a sporotrichosis epidemic in Rio de Janeiro, Brazil. Int. J. Gynaecol. Obs..

[277] Legabão B.C., Fernandes J.A., de Oliveira Barbosa G.F., Bonfim-Mendonça P.S., Svidzinski T.I.E. (2022). The zoonosis sporotrichosis can be successfully treated by photodynamic therapy: A scoping review. Acta Trop..

[278] Borba-Santos L.P., Gagini T., Ishida K., de Souza W., Rozental S. (2015). Miltefosine is active against *Sporothrix brasiliensis* isolates with in vitro low susceptibility to amphotericin B or itraconazole. J. Med. Microbiol..

[279] Fischman Gompertz O., Rodrigues A.M., Fernandes G.F., Bentubo H.D., de Camargo Z.P., Petri V. (2016). Atypical clinical presentation of sporotrichosis caused by *Sporothrix globosa* resistant to itraconazole. Am. J. Trop. Med. Hyg..

[280] Nakasu C.C.T., Waller S.B., Ripoll M.K., Ferreira M.R.A., Conceição F.R., Gomes A.D.R., Osório L.D.G., de Faria R.O., Cleff M.B. (2021). Feline sporotrichosis: A case series of itraconazole-resistant *Sporothrix brasiliensis* infection. Braz. J. Microbiol..

[281] Waller S.B., Dalla Lana D.F., Quatrin P.M., Ferreira M.R.A., Fuentefria A.M., Mezzari A. (2021). Antifungal resistance on *Sporothrix* species: An overview. Braz. J. Microbiol..

[282] Lyra M.R., Sokoloski V., de Macedo P.M., Azevedo A.C.P. (2021). Sporotrichosis refractory to conventional treatment: Therapeutic success with potassium iodide. An. Bras. De Dermatol..

[283] Poester V.R., Basso R.P., Stevens D.A., Munhoz L.S., de Souza Rabello V.B., Almeida-Paes R., Zancopé-Oliveira R.M., Zanchi M., Benelli J.L., Xavier M.O. (2022). Treatment of human sporotrichosis caused by *Sporothrix brasiliensis*. J. Fungi.

[284] Bernardes-Engemann A.R., Tomki G.F., Rabello V.B.d.S., Almeida-Silva F., Freitas D.F.S., Gutierrez-Galhardo M.C., Almeida-Paes R., Zancopé-Oliveira R.M. (2022). Sporotrichosis caused by non-wild type *Sporothrix brasiliensis* strains. Front. Cell. Infect. Microbiol..

[285] Lee Y., Puumala E., Robbins N., Cowen L.E. (2021). Antifungal drug resistance: Molecular mechanisms in *Candida albicans* and beyond. Chem. Rev..

[286] Koehler A., Pagani D.M., da Silva Hellwig A.H., Scroferneker M.L. (2021). In-vitro antifungal susceptibility of the genus *Sporothrix* and correlation with treatment options for sporotrichosis: A systematic review. Rev. Med. Microbiol..

[287] Gagini T., Borba-Santos L.P., Messias Rodrigues A., Pires de Camargo Z., Rozental S. (2017). Clotrimazole is highly effective in vitro against feline *Sporothrix brasiliensis* isolates. J. Med. Microbiol..

[288] Fichman V., Marques de Macedo P., Francis Saraiva Freitas D., Carlos Francesconi do Valle A., Almeida-Silva F., Reis Bernardes-Engemann A., Zancopé-Oliveira R.M., Almeida-Paes R., Clara Gutierrez-Galhardo M. (2021). Zoonotic sporotrichosis in renal transplant recipients from Rio de Janeiro, Brazil. Transpl. Infect. Dis..

[289] Georgopoulos A., Petranyi G., Mieth H., Drews J. (1981). *In vitro* activity of naftifine, a new antifungal agent. Antimicrob. Agents Chemother..

[290] Artunduaga Bonilla J.J., Honorato L., Haranahalli K., Gremião I.D.F., Pereira S.A., Guimarães A., Baptista A.R.S., Patricia d.M.T., Rodrigues M.L., Miranda K. (2021). Antifungal activity of Acylhydrazone derivatives against *Sporothrix* spp.. Antimicrob. Agents Chemother..

[291] Borba-Santos L.P., Visbal G., Braga T.G., Rodrigues A.M., De Camargo Z.P., Bezerra L.L., Ishida K., De Souza W., Rozental S. (2016). ∆24-sterol methyltransferase plays an important role in the growth and development of *Sporothrix schenckii* and *Sporothrix brasiliensis*. Front. Microbiol..

[292] Borba-Santos L.P., Nucci M., Ferreira-Pereira A., Rozental S. (2021). Anti-*Sporothrix* activity of ibuprofen combined with antifungal. Braz. J. Microbiol..

[293] Borba-Santos L.P., Ishida K., Calogeropoulou T., Souza W., Rozental S. (2016). Adamantylidene-substituted alkylphosphocholine TCAN26 is more active against Sporothrix schenckii than miltefosine. Mem. Do Inst. Oswaldo Cruz.

[294] Asquith C.R.M., Machado A.C.S., de Miranda L.H.M., Konstantinova L.S., Almeida-Paes R., Rakitin O.A., Pereira S.A. (2019). Synthesis and identification of pentathiepin-based inhibitors of *Sporothrix brasiliensis*. Antibiotics.

[295] Gopinath P., Yadav R.K., Shukla P.K., Srivastava K., Puri S.K., Muraleedharan K.M. (2017). Broad spectrum anti-infective properties of benzisothiazolones and the parallels in their anti-bacterial and anti-fungal effects. Bioorg. Med. Chem. Lett..

[296] Azevedo-França J.A., Granado R., de Macedo Silva S.T., Santos-Silva G.D., Scapin S., Borba-Santos L.P., Rozental S., de Souza W., Martins-Duarte É S., Barrias E. (2020). Synthesis and biological activity of novel zinc-itraconazole complexes in protozoan parasites and *Sporothrix* spp.. Antimicrob. Agents Chemother..

[297] de Azevedo-França J.A., Borba-Santos L.P., de Almeida Pimentel G., Franco C.H.J., Souza C., de Almeida Celestino J., de Menezes E.F., Dos Santos N.P., Vieira E.G., Ferreira A. (2021). Antifungal promising agents of zinc(II) and copper(II) derivatives based on azole drug. J. Inorg. Biochem..

[298] Gagini T., Colina-Vegas L., Villarreal W., Borba-Santos L.P., de Souza Pereira C., Batista A.A., Kneip Fleury M., de Souza W., Rozental S., Costa L.A.S. (2018). Metal–azole fungistatic drug complexes as anti-*Sporothrix* spp. agents. New J. Chem..

[299] Tandon V.K., Kumar S., Mishra N.N., Shukla P.K. (2012). Micelles catalyzed chemo- and regio-selective one pot and one step synthesis of 2,3,5,6-tetrakis(alkyl and arylsulfanyl)-1,4-benzoquinones and 2,5-diaminosubstituted-1,4-benzoquinones “In-Water” and their biological evaluation as antibacterial and antifungal agents. Eur. J. Med. Chem..

[300] Brilhante R.S., Silva N.F., Marques F.J., Castelo-Branco Dde S., de Lima R.A., Malaquias A.D., Caetano E.P., Barbosa G.R., de Camargo Z.P., Rodrigues A.M. (2015). In vitro inhibitory activity of terpenic derivatives against clinical and environmental strains of the *Sporothrix schenkii* complex. Med. Mycol..

[301] Brilhante R.S., Malaquias A.D., Caetano E.P., Castelo-Branco Dde S., Lima R.A., Marques F.J., Silva N.F., Alencar L.P., Monteiro A.J., Camargo Z.P. (2014). In vitro inhibitory effect of miltefosine against strains of *Histoplasma capsulatum var. capsulatum* and *Sporothrix* spp.. Med. Mycol..

[302] Borba-Santos L.P., Vila T., Rozental S. (2020). Identification of two potential inhibitors of *Sporothrix brasiliensis* and *Sporothrix schenckii* in the Pathogen Box collection. PLoS ONE.

[303] Borba-Santos L.P., Barreto T.L., Vila T., Chi K.D., Dos Santos Monti F., de Farias M.R., Alviano D.S., Alviano C.S., Futuro D.O., Ferreira V. (2021). In vitro and in vivo antifungal activity of buparvaquone against *Sporothrix brasiliensis*. Antimicrob. Agents Chemother..

[304] Brilhante R.S., Pereira V.S., Oliveira J.S., Lopes R.G., Rodrigues A.M., Camargo Z.P., Pereira-Neto W.A., Castelo-Branco D.S., Cordeiro R.A., Sidrim J.J. (2018). Pentamidine inhibits the growth of *Sporothrix schenckii* complex and exhibits synergism with antifungal agents. Future Microbiol..

